# Immunoglobulin G *N*-Glycome as a biomarker of mortality risk in *Escherichia coli* induced sepsis

**DOI:** 10.3389/fimmu.2025.1532145

**Published:** 2025-03-17

**Authors:** Huachen Wang, Houqiang Li, Zheng Guo, Hongda Hou, Haifeng Hou, Bing Chen

**Affiliations:** ^1^ Institute of Infectious Diseases, The Second Hospital of Tianjin Medical University, Tianjin, China; ^2^ Intensive Care Unit, The Second Hospital of Tianjin Medical University, Tianjin, China; ^3^ Shandong Provincial Third Hospital, Cheeloo College of Medicine, Shandong University, Jinan, China; ^4^ School of Public Health, Shandong First Medical University & Shandong Academy of Medical Sciences, Jinan, China; ^5^ Centre for Precision Health, School of Medical and Health Sciences, Edith Cowan University, Perth, WA, Australia

**Keywords:** IgG glycosylation, mortality, *Escherichia coli*, sepsis, biomarker

## Abstract

**Background:**

Sepsis is a life-threatening syndrome caused by an imbalance in the inflammatory response to an infection that can lead to a high mortality rate. *Escherichia coli* is a common pathogen that causes sepsis. The role of immunoglobulin G *N*-glycome in estimating the mortality in patients with sepsis remains unknown. This study aims to reveal the clinical application of immunoglobulin G *N*-glycome as a potentially novel biomarker to predict mortality risk in *Escherichia coli*-induced sepsis.

**Methods:**

The serum immunoglobulin G *N*-glycome levels in 100 adult septic patient serum samples on the day of intensive care unit (ICU) admission, and 100 healthy volunteers were measured and analyzed. Immunoglobulin G *N*-glycome was compared with existing risk scores on predicting in-hospital death.

**Results:**

We identified that the fucosylation level was significantly decreased in patients. Importantly, bisecting GlcNAc, sialylation, and galactosylation have different levels between sepsis and control groups. In addition, the AUC values of the SOFA score combined with GP4, GP5, and GP9 were 0.76 (95%CI: 0.61 to 0.90), 0.58 (95%CI: 0.40 to 0.7) and 0.57 (95%CI: 0.38 to 0.76). The AUC value of the SOFA score combined with GP4 and GP7 was 0.85 (95%CI: 0.76 to 0.93) in predicting in-hospital mortality in patients with sepsis.

**Conclusions:**

Immunoglobulin G *N*-glycome concentrations at ICU admission are valuable for predicting the in-hospital mortality risk of patients with sepsis, suggesting that immunoglobulin G *N*-glycome may be a novel biomarker.

## Introduction

Sepsis is characterized by a dysregulated host immune response to infection (1, 2). It can damage multiple organs, including the heart, brain, kidneys, and liver, leading to septic shock and organ failure (3). Sepsis is the leading cause of death in hospitals and intensive care units, with mortality rates ranging from 10% to 50% depending on the severity of the infection and the overall health of the patient (4). When the immune system is compromised or suppressed, it is harder for the patient to fight off invading bacteria, and causes hyperinflammation (5, 6). *Escherichia coli* is a common pathogen that causes sepsis. Circulating immunoglobulin (Ig) G is an important immune effector that protects organisms from a wide variety of bacteria (7). Alterations in IgG-associated glycans can influence the resistance of mice to sepsis by affecting the function of IgG effectors (8). However, little is known about the glycosylation profile of IgG during pathogen invasion.

Glycosylation is a common and important post-translational modification that can affect protein stability, and function and is involved in almost all pathophysiological processes (7). In healthy individuals, the glycan phenotype is stable but changes significantly under pathological conditions such as inflammation (9, 10). IgG is the most abundant glycoprotein in human biological fluids and a major effector of the humoral immune system (11). IgG is involved in inflammatory pathways primarily through its *N*-glycans *(* 12, 13). The *N*-glycans of IgG can completely alter its anti-inflammatory and pro-inflammatory activity (14). Studies have shown that IgG *N*-glycan changes are involved in the pathogenesis of cardiovascular diseases (15), cancer (16), and autoimmune diseases (17).

However, IgG *N*-glycome has been rarely studied in patients with sepsis. Here, we undertook IgG *N*-glycome profiling in blood samples from health volunteers and patients with sepsis. In the present study, we try to reveal the relationship between IgG *N*-glycome and the prognosis in patients with sepsis, meanwhile, we explore whether the serum IgG N-Glycome concentration at the time of ICU admission can be used as a potential biomarker for predicting mortality risk in *Escherichia coli*-induced sepsis.

## Methods

### Data source

The study adopted a case-control study design, including 100 *Escherichia coli*-induced septic patients and 100 healthy controls. All study subjects were individuals enrolled in the Second Hospital of Tianjin Medical University.

This protocol was approved by the Clinical Research Ethics Committee of the Second Hospital of Tianjin Medical University (KY2022K234), and informed consent was conformed to the Declaration of Helsinki (18). The consent of each participant was obtained at admission. If the patient has lost consciousness or capacity, written informed consent was then obtained from each enrolled patient’s nearest relative or designated person.

Septic patients were eligible if they met the following criteria: (1) were aged 18 years or older, (2) met clinically diagnosed criteria for sepsis according to the third international consensus definitions (Sepsis-3) (19), (3) were hospitalized and had serum samples, and (4) *Escherichia coli* induced sepsis and were not involved in the other clinical trial.

Septic patients were excluded if they had one of the following criteria: (1) no available clinical data, (2) women who are pregnant or breastfeeding, (3) non-septic inflammatory or other non-infection related diagnoses causing organ dysfunction (severe trauma or burns, immunopathy or neoplasm), (4) patients who had an unclear baseline Sequential Organ Failure Assessment (SOFA) score, and (5) sequential organ failure assessment (SOFA) score < 2.

Healthy controls were subjects who underwent physical examination in the hospital and whose plasma samples were retained. All study participants signed informed consent forms. Moreover, informed consent was obtained from family members of unconscious patients.

### Study Measurements and Procedures

At enrolment, patients and controls were interviewed to obtain general demographic characteristics and epidemiological information. The clinical records were reviewed to determine information on treatment, and physiological and biochemical indicators.

### Plasma sample collection

5 ml fasting blood samples were collected by nursing staff into heparin vacutainer blood collection tubes. Samples were separated by centrifugation and the supernatant was transferred to 1.5 ml cryovials after processing. All patients and control provided at least 200 μL of plasma samples, which were stored at minus 80 degrees Celsius until analyzed in the laboratory.

### Analysis of IgG glycans

Laboratory isolation, release, and testing for IgG *N*-glycome composition were performed as described previously (20, 21). Briefly, 100 μL plasma sample diluted by 700 μL 1× phosphate buffer saline (PBS, PH = 7.4) was transferred into Protein G 0.2 mL Monolithic 96-well Plate (BIA Separation, European Union), and then IgG glycans were washed with 1 ml of 0.1 M formic acid and neutralized with 1 M ammonium bicarbonate.

30 μL 1.33% SDS (w/v) (Invitrogen, Carlsbad, CA, USA) was added into the dried IgG-powder, and IgG *N*-glycan was released with 1 μL PNGase F (Glycerol-free) at 37 degrees Celsius for 18 hours.

The free glycans were labeled with 2-aminobenzamide (2-AB). Enzymatically released IgG *N*-glycans were separated by Hydrophilic interaction chromatography-ultra performance liquid chromatography (HILIC-UPLC) on Waters Acquity UPLC instrument (Waters, Milford, MA, USA).

The UPLC chromatogram was analyzed using the automated image processing software with a traditional integration algorithm after which the manual adjustment was performed to ensure the accuracy of 24 directly measured glycan peaks. The amount of each glycans peak was calculated as the percentage of the total integrated spectral ranges covering. Moreover, 51 derived glycan traits were calculated with 24 directly measured glycans.

### Statistical analysis

Categorical variables were presented as frequency and percent and the Pearson’s chi-squared test (χ ^2^ test) was performed. Continuous variables were evaluated using the Kolmogorov-Smirnov normality test, and mean ± standard deviation (SD) or median and interquartile range (IQR) were described according to the normality of distribution. Furthermore, one-way analysis of variance (ANOVA) was performed for continuous variables with normal distribution, and the Mann-Whitney U was used to compare two groups for those with normal distribution.

The whole original samples were randomly divided into training and validation sets according to the ratio of 7:3 to verify the stability of the model. The least absolute shrinkage and selection operator (LASSO) regularization technique and recursive feature elimination (RFE) were used to pick out each robust IgG *N*-linked glycan associated with the disease because of the high sensitivity of the lasso and RFE for the variations of multiple independent variables (22, 23). The multivariate logistic regression model was fitted in the analysis, which was coded as a binary variable with filtered variables, and subsequently, the models adjusted for age, sex, fasting blood glucose (FBG), uric acid (UA), alanine transaminase (ALT), aspartate aminotransferase (AST), and cerebral-cardio vascular diseases (CVD). Coefficients and odds ratios (ORs) with their 95% confidence intervals (CIs) are also presented. Moreover, the final model included significant variables that were used to construct the receiver operation characteristic (ROC) curve, and the area under the curve (AUC), sensitivity, and specificity were calculated. In our analysis, R software (version 4.2.3) and SPSS (version 26.0) were used to calculate. R package ‘glmnet’ (version 0.6-3) was used to perform the lasso regression. Two-sided *p* values of 0.05 or lower were deemed to be significant.

## Results

### Characteristics of study participants

We included 100 healthy adults (63 women and 37 men, 58.32 ± 0.59 years) and 100 patients with *Escherichia coli*-induced sepsis (48 women and 52 men), with an average age of 68.94 ± 1.47 years. The demographic characteristics of the study populations are presented in **Table 1**. The median FBG, UA, ALT, and AST levels in septic patients were also significantly higher than in the control group. The sepsis group had a higher prevalence of CVD compared to the other groups. Among the 100 patients with sepsis during their hospitalization, 16 died. The in-hospital mortality was 16%.

**Table 1:** Characteristics of the study participants.

Variables	Controls (n=100)	Sepsis (n=100)	*t/Z*/χ^2^	*P*
Age	58.32 ± 0.59	68.94 ± 1.47	-6.72^a^	1.93E-10
Sex (n, %)			-2.12^b^	0.03
Male	37 (37%)	52 (52%)		
Female	63 (63%)	48 (48%)		
SOFA	NA	8.05 ± 3.03		
FBG (mmol/L)	5.92 (5.45, 6.30)	9.205 (6.59, 10.78)	-7.43^c^	1.11E-13
UA (µmol/L)	288.00 (231.50, 345.00)	435.45 (267.37, 566.40)	-5.45^c^	5.00E-08
ALT (U/L)	20.15 (14.73, 23.78)	30.65 (19.23, 62.15)	-5.31^c^	1.12E-07
AST (U/L)	21.85 (18.93, 25.95)	40.15 (25.00, 112.00)	-6.93^c^	4.18E-12
WBC (10*9/L)	NA	18.10 (11.39, 22.75)		
PLT (10*9/L)	NA	187.00 (107.00, 273.00)		
CVD (n, %)			29.48^b^	1.34E-21
Presence	4 (4.0%)	31 (31%)		
Absence	96 (96%)	69 (69%)		
Number of deaths	NA	16 (16%)		

SOFA, Sequential Organ Failure Assessment; FBG, Fasting Blood Glucose; UA, Uric Acid; ALT, Alanine Transaminase; AST, Aspartate Aminotransferase; WBC, White Blood Cell; PLT, Platelets; CVD, Cerebral-cardio Vascular Diseases; Not Available; ^a^T-test; ^b^Pearson’s chi-squared test. ^c^Mann-Whitney U Test.

### The IgG glycome composition among different groups of people

**Figure 1:**
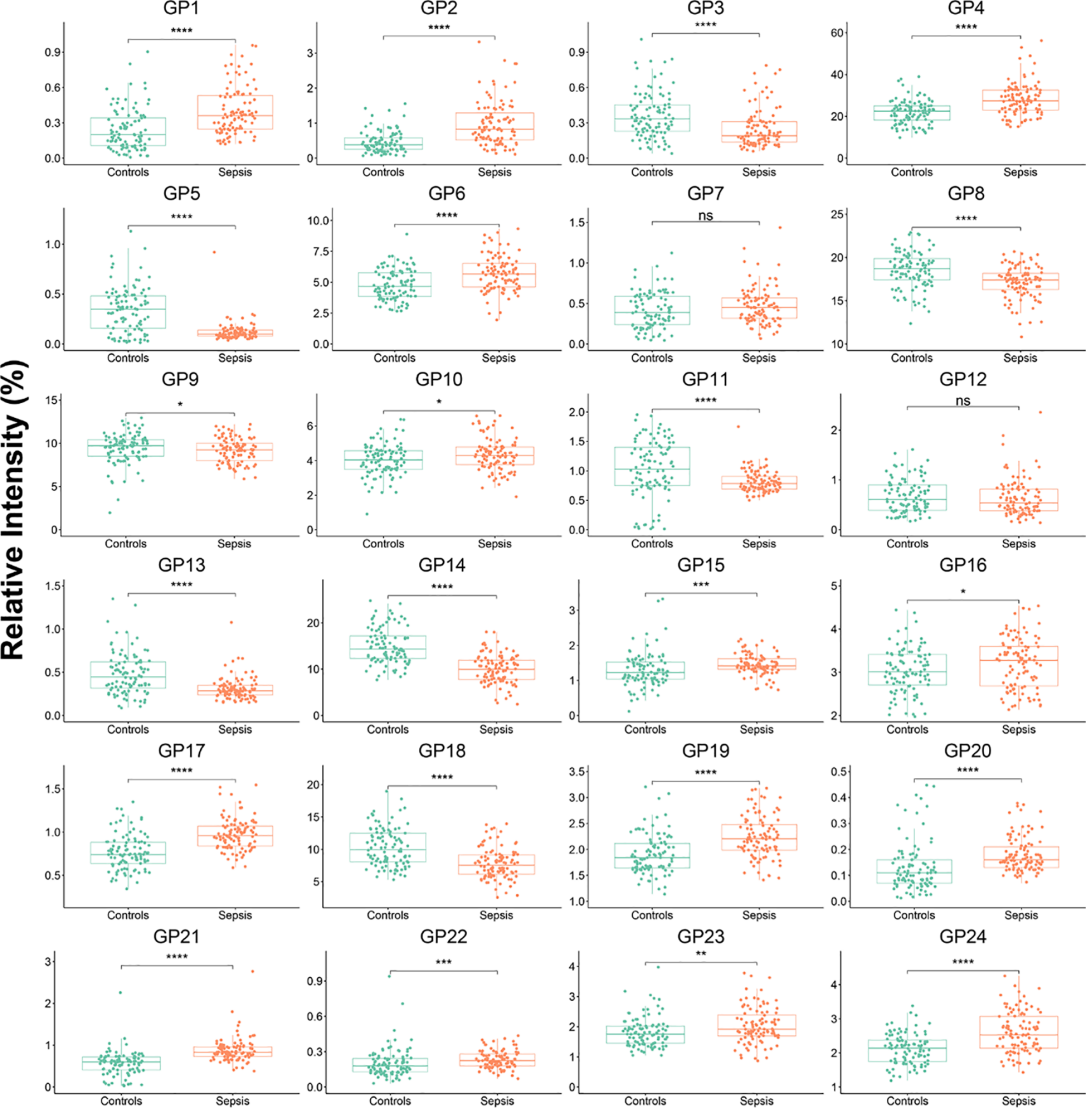
IgG initial *N*-glycans in controls and septic patients. Mann-Whitney U Test was used. *p<0.05, **p<0.01, ***p<0.001, ****p<0.0001; ns means not significant.

We measured the 24 plasma IgG glycome composition and calculated the 51 deprived glycan traits between controls and sepsis groups by a previously established method (24). There were 22 initial glycans and 38 deprived glycans that were significantly different between the two groups (**Figure 1**; **Supplementary Figures S1-S5**, **Supplementary Tables S1, S2**). No statistical difference was observed among 2 initial glycans (GP7 and GP12) and 13 deprived glycans (GP7n, GP9n, GP10n, GP12n, FGS/(FG+FGS), FBG2S1/(FBG2+FBG2S1+FBG2S2), FBG2S2/(FBG2+FBG2S1+FBG2S2), FBS1/FBS2, FBS2/FS2, FBS2/(FS2+FBS2) FBS2/(FS2+FBS2), Fn total, FG0n/G0n and BG2n/(FG2n + FBG2n)).

#### Fucosylation

As illustrated in **Table 2**, the fucosylation level was significantly decreased in patients (sepsis: 95.09%) compared with the control population (95.83%).

**Table 2:** The main IgG *N-*glycans in controls and septic patients.

Glycans	Controls (n=100)	Sepsis (n=100)	*Z*	*P*
Fucosylation	95.83 (94.89, 96.43)	95.09 (94.19, 96.03)	-3.11	1.89E-03
Bisecting GlcNAc	15.86 (14.33, 17.40)	17.38 (15.89, 19.50)	-4.68	2.86E-06
Sialylation	21.70 (18.67, 23.93)	19.57 (17.88, 22.39)	-2.61	0.01
Galactosylation				
G0	28.24 (23.35, 32.03)	35.04 (29.00, 41.17)	-6.26	3.79E-10
G1	34.59 (32.58, 35.87)	32.40 (30.59, 33.70)	-5.05	4.38E-07
G2	17.21 (14.08, 20.51)	12.44 (9.88, 14.48)	-7.81	5.54E-15

G0, agalactosylation; G1, monogalactosylation; G2, digalactosylation; Mann-Whitney U Test was used.

#### Bisecting GlcNAc

The level of *N*-glycan with bisecting GlcNAc in patients with sepsis (17.38%) was significantly higher than in the control (15.86%) groups.

#### Sialylation

The percentage of glycans with sialylation was 19.57% in the septic patients, which was significantly lower than that in the control (21.70%) groups.

#### Galactosylation

The agalactosylated *N*-glycans of septic patients (35.04%) were significantly higher than those of controls (28.24%). It was also observed that, in the human plasma, monogalactosylated *N*-glycans were distinctively decreased in septic patients (32.40%) relative to the control group (34.59%). In addition, lower digalactosylated *N*-glycans were found in septic patients (12.44%) than in the controls (17.21%).

### Discrimination of the septic patients using IgG initial *N*-glycans

**Figure 2:**
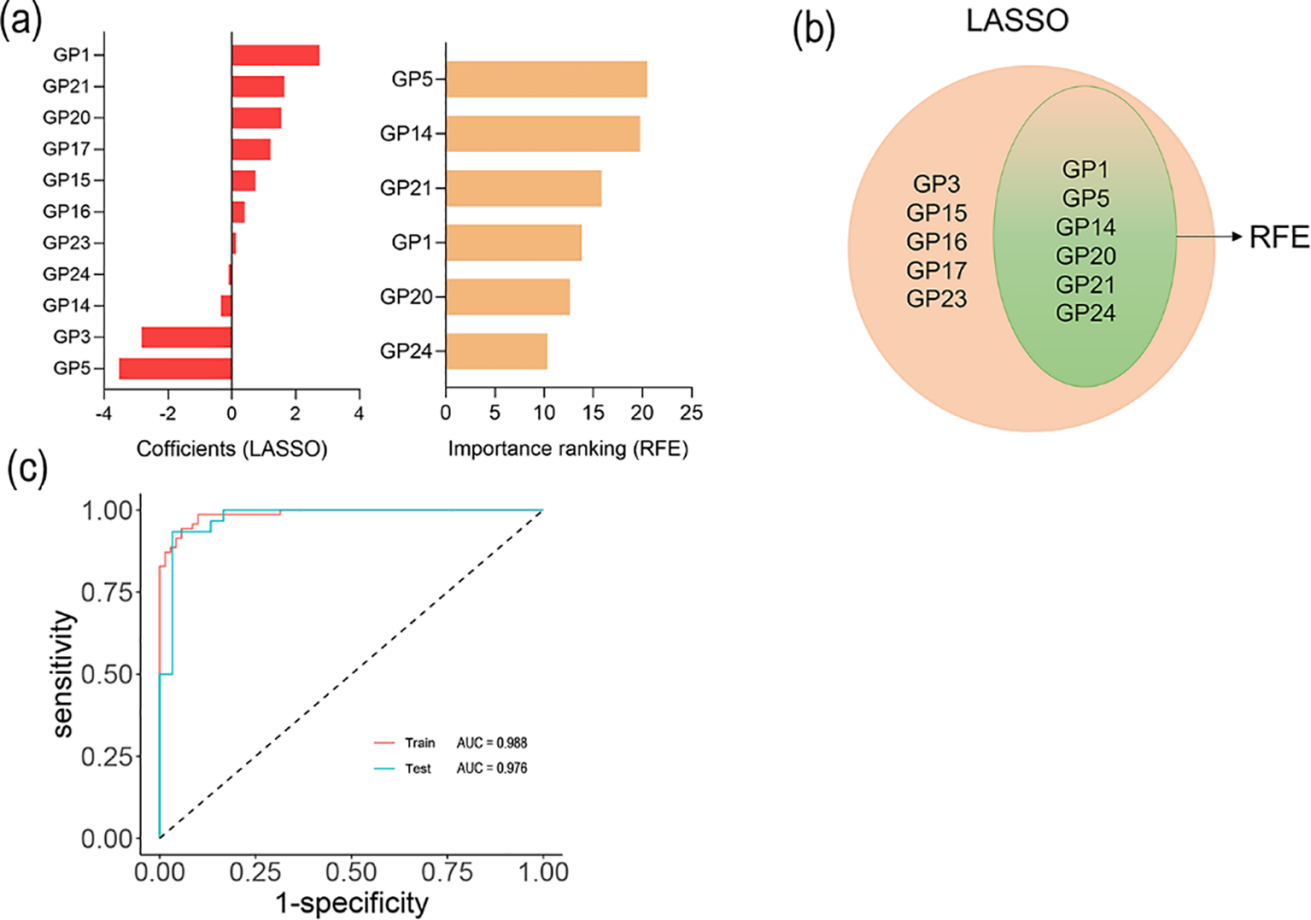
Selected IgG N-glycans and ROC curves. **(a)** Coefficient of LASSO and RFE algorithms; **(b)** Variable coincidence; **(c)** ROC curves of logistic regression model among training and testing sets.

To evaluate the combined effects of 24 initial *N*-glycans on sepsis, we fitted the disease diagnostic model in training sets. Firstly, we used the LASSO and RFE algorithms to filter 24 initial *N*-glycans and optimize the complexity of the model, identifying 6 initial *N*-glycans (GP1, GP5, GP14, GP20, GP21 and GP24) for sepsis (**Figures 2a, b**). What’s more, we performed a binary classification logistic regression (**Table 3**). It was found that, after further adjustment for age, sex, FBG, UA, ALT, AST, and CVD, two glycans (GP5 and GP14) were negatively associated with sepsis, and two glycans (GP21 and GP22) were positively associated with sepsis. Furthermore, the model of combined *N*-glycans showed a good diagnostic performance in the internal training set (AUC: 0.988, 95%CI: 0.945 to 1.000) (**Figure 2c**). Similarly, the AUC value of combined *N*-glycans for diagnosing sepsis in the testing set can reach up to 0.976 (95%CI: 0.940 to 1.000) (**Figure 2c**). The further filtering process of the LASSO and RFE algorithms is outlined in **Supplementary Figures S6, S7**.

**Table 3:** Logistic models of the initial IgG *N-*glycans in septic patients.

Variables	*β*	SE	Walds	OR (95% CI)	*P*
GP5	-10.02	3.19	9.90	4.44E-05 (8.63E-08, 0.02)	1.65E-03
GP14	-0.38	0.14	7.13	0.69 (0.52, 0.91)	0.01
GP21	4.04	1.45	7.75	56.63 (3.30, 9.71E+02)	0.01
GP24	0.73	0.31	5.47	2.07 (1.13, 3.82)	0.02
FBG	0.57	0.20	8.49	1.77 (1.21, 2.60)	3.56E-03
CVD	3.92	1.18	11.10	50.18 (5.01, 5.02E+02)	8.63E-04

*β*, Standardized Regression Coefficient; SE, Standard Error; OR, Odds Ratio; CI, Confidence Interval; CVD, Cerebral-cardio Vascular Diseases.

The multivariate logistic regression was performed after LASSO and RFE; Adjusted for age, sex, FBG, UA, ALT, AST and CVD.

### Difference of the IgG glycome composition between survivor and non-survivor in patients with sepsis during hospitalization

**Figure 3:**
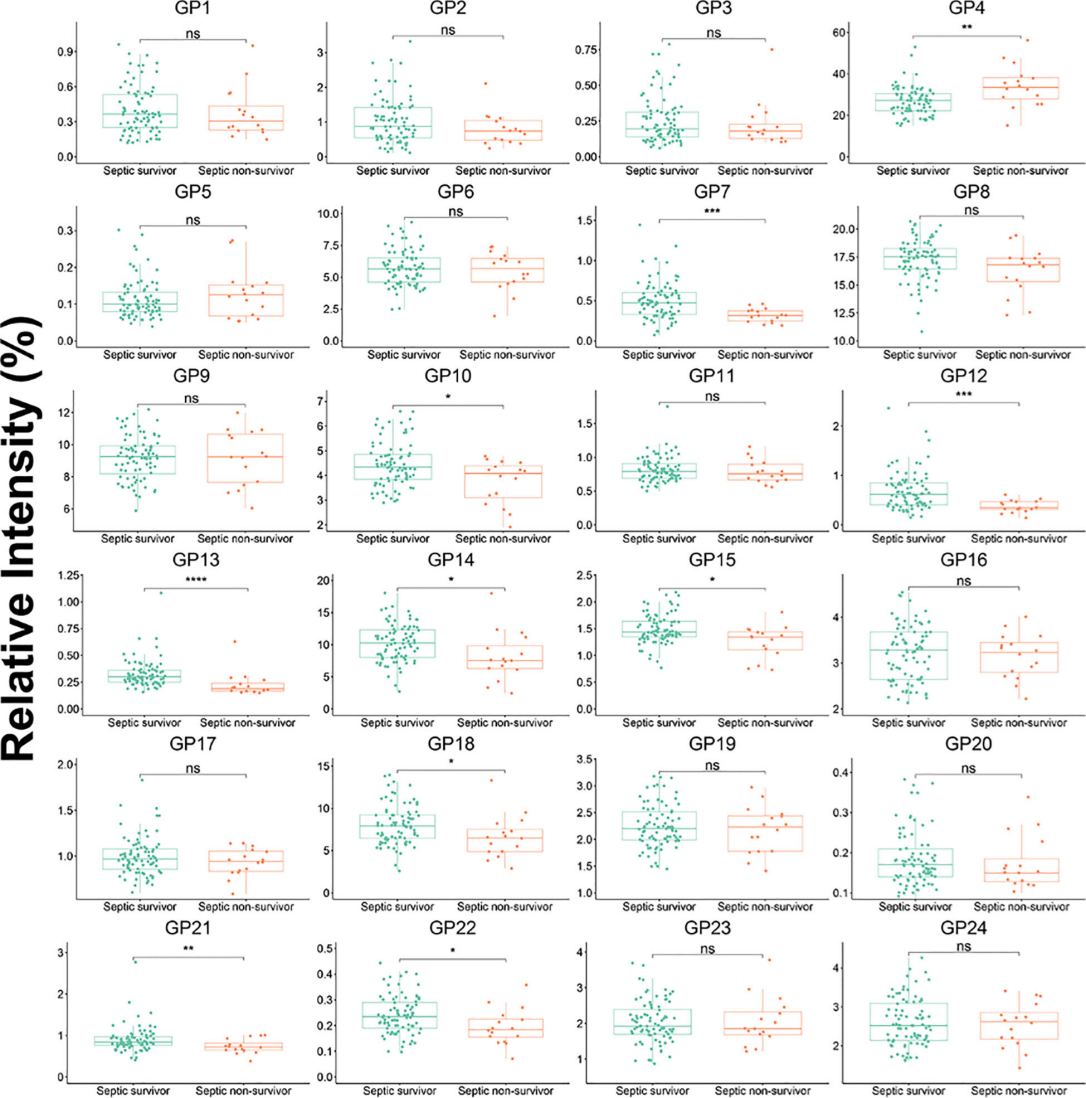
IgG initial N-glycans in septic survivor and septic non-survivor during hospitalization. Mann-Whitney U Test was used. *p<0.05, **p<0.01, ***p<0.001, ****p<0.0001; ns means not significant.

A statistically significant difference in 10 initial IgG *N*-glycans (GP4, GP7, GP10, GP12, GP13, GP14, GP15, GP18, GP21, and GP22) and 25 deprived IgG *N*-glycans was observed between non-surviving septic patients and surviving patients (**Figure 3**; **Supplementary Table S3**). The level of sialylation, G1, and G2 in septic non-survivor was 20.10%, 32.66%, and 12.98%, respectively, which was significantly higher than in septic survivors. The level of fucosylation (94.97%) and G0 (34.58%)in septic non-survivor was significantly lower than in septic survivors.

### Subgroup analysis on gender

Subgroup analysis on gender was conducted to explore the changes of IgG *N*-glycan (**Supplementary Table S4**).The finding suggested that there were no significant difference in IgG *N-*glycome levels of Fucosylation, Bisecting GlcNAc, Sialylation, and Galactosylation.

### The impact of IgG *N*-glycans on mortality during hospitalization in septic patients

**Figure 4:**
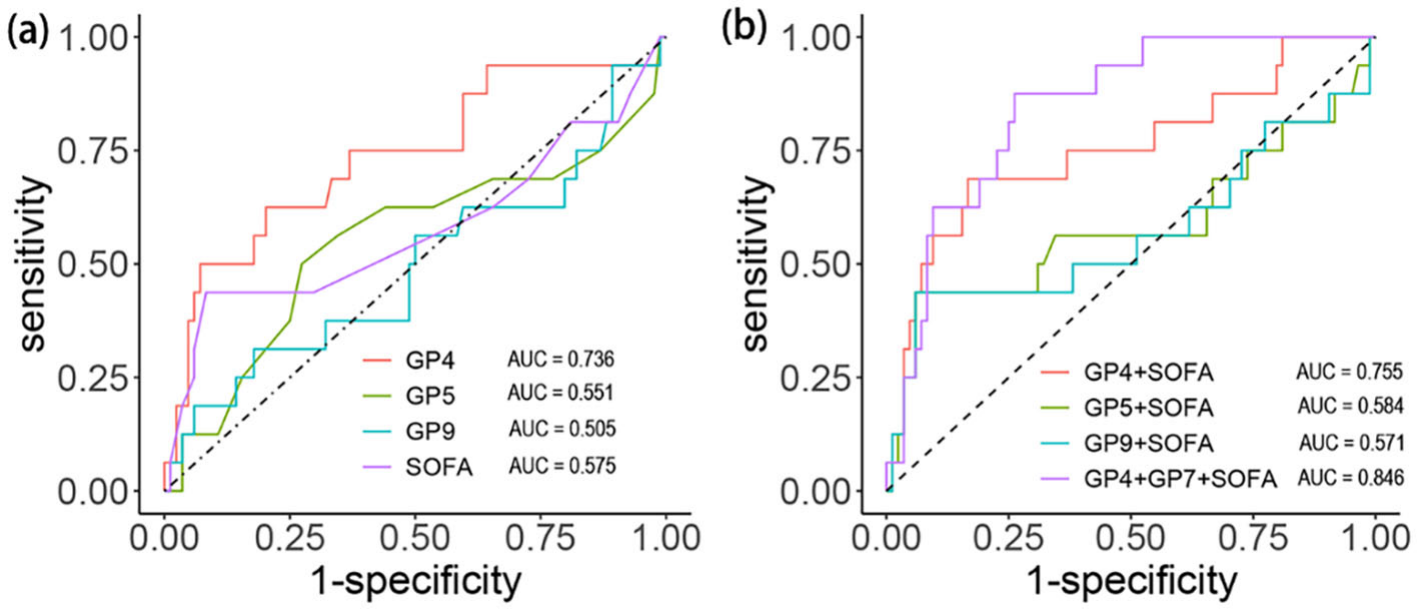
Discrimination capacity of initial glycans and SOFA score for in-hospital death among septic patients. **(a)** Efficacy of each indicator. **(b)** Efficacy of combined indicators.

We calculated the AUC value, SE, SP, positive predictive value (PPV), and negative predictive value (NPV) to evaluate the predictive power of different indicators and draw the ROC curve (**Table 4**, **Figure 4a**). The AUC values of SOFA, GP4, GP5, and GP9 were 0.58 (95%CI: 0.39to 0.76), 0.74 (95%CI: 0.59 to 0.86), 0.55 (95%CI: 0.37 to 0.73) and 0.51 (95%CI: 0.33 to 0.68), respectively. Furthermore, we further evaluated the performance of the SOFA score combined with IgG *N*-glycans to predict in-hospital mortality (**Table 5**, **Figure 4b**). The AUC values of the SOFA score combined with GP4, GP5, and GP9 were 0.76 (95%CI: 0.61 to 0.90), 0.58 (95%CI: 0.40 to 0.7) and 0.57 (95%CI: 0.38 to 0.76). The AUC value of the SOFA score combined with GP4 and GP7 was 0.85 (95% CI: 0.76 to 0.93).

**Table 4:** The AUC and the other parameters in SOFA and IgG *N-*glycans for mortality during the hospitalization in septic patients.

Variable	AUC	95%LCI	95%UCI	Cut-off value	SE (%)	SP (%)	PPV (%)	NPV (%)
GP4	0.74	0.59	0.86	34.19	0.50	0.93	0.77	0.80
GP5	0.55	0.37	0.73	0.13	0.50	0.73	0.46	0.76
GP9	0.51	0.33	0.68	7.72	0.31	0.82	0.45	0.72
SOFA	0.58	0.39	0.76	10.50	0.44	0.92	0.71	0.78

AUC, Area Under the Curve; LCI, Lower Confidence interval; UCI, Upper Confidence interval; Cut-off value, the optimal cutoff points for mortality during the hospitalization; SE, sensitivity; SP, specificity; PPV, positive predictive value; NPV, negative predictive value.

**Table 5:** The AUC and the other parameters in the uniting variable for morality during the hospitalization in septic patients.

Variable	AUC	95%LCI	95%UCI	SE (%)	SP (%)	PPV (%)	NPV (%)
GP4+SOFA	0.76	0.61	0.90	0.69	0.83	0.66	0.85
GP5+SOFA	0.58	0.40	0.77	0.44	0.94	0.78	0.78
GP9+SOFA	0.57	0.38	0.76	0.44	0.94	0.78	0.78
GP4+GP7+SOFA	0.85	0.76	0.93	0.88	0.74	0.61	0.93

AUC, Area under the curve; LCI, Lower Confidence interval; UCI, Upper Confidence interval; SE, sensitivity; SP, specificity; PPV, positive predictive value; NPV, negative predictive value.

### Independent predictors of in-hospital mortality

In binary logistic regression analysis, GP4 (*P* = 9.08E-05) and SOFA (*P* = 7.99E-04) were significantly associated with in-hospital mortality, whereas GP5 (OR: 2.65, [95% CI: 0.89-7.90]; *P* = 0.08) and GP9 (OR: 0.48, [95% CI: 0.14-1.58]; *P* = 0.23) were not (**Table 6**). The higher lever of GP4 was 13 times higher risk of death and the higher lever of SOFA score increased the risk of death 8.56 times.

**Table 6:** The independent predictors of morality in hospitalization.

Variables	*β*	SE	Walds	OR (95% CI)	*P*
GP4	2.56	0.66	15.32	13.00 (3.60, 46.97)	9.08E-05
GP5	0.98	0.56	3.07	2.65 (0.89, 7.90)	0.08
GP9	-0.74	0.61	1.46	0.48 (0.14, 1.58)	0.23
SOFA	2.15	0.64	11.24	8.56 (2.44, 30.00)	7.99E-04

SOFA, Sequential Organ Failure Assessment; *β*, standardized regression coefficient; SE, Standard Error; OR, Odds Ratio; CI, Confidence Interval; The concentrations of GP4, GP5, GP9 and SOFA were judged to be the negative (= 0) and positive (= 1) according to the cut-off point value (GP4, 34.19; GP5, 0.13;GP9, 7.72;SOFA, 10.50).

## Discussion

The high mortality rate of sepsis imposes a huge burden on society (4). At present, there are no reliable biomarkers for predicting the prognosis of patients with sepsis upon admission to the ICU (25). Serum IgG *N*-glycome are new indicators of human infectious diseases (26). We found that serum IgG *N*-glycome may be effective markers for diagnosing and predicting the mortality risk of patients with sepsis. First, a large number of the 24 plasma IgG glycome among patients with sepsis were significantly different from those of healthy controls. Second, GP4 in IgG glycome is associated with sepsis outcomes. The serum GP4 level of non-surviving patients with sepsis was higher than that of the surviving ones on the day of admission. Third, GP4 is better than other indicators (such as SOFA) in predicting septic patients’ in-hospital mortality. In addition, the combination of serum GP4 with other study measures (such as GP5 and GP9) in patients with sepsis significantly improved the prediction of the risk of death in the hospital. Fourth, serum GP4 and SOFA scores on the day of ICU admission in patients with bacterial sepsis were independent predictors of in-hospital mortality. The risk of mortality in patients with sepsis and high serum GP4 concentration was 13.0 times that of patients with sepsis and low serum concentration. Finally, according to the survival curve analysis, patients with high serum GP4 admission level have poorer survival rates than patients with low serum GP4 admission level.

The essence of sepsis is that infection causes excessive inflammation and immune suppression (27). Previous studies have also shown that IgG is the most common Ig circulating in the blood, and IgG *N*-glycans is the most well-understood character of IgG, especially in the context of infection (28). These glycans affect antibody secretion, half-life, stability, immunogenicity, and effector function (29). Differences in IgG *N*-glycans profiles between patients infected with different pathogens are significant and reflect genetic and environmental factors (30), which may lead to abnormal expression of glycosyltransferase and glycosidase in immune cells, resulting in different susceptibility to pathogen infection. The level of total IgG galactosylation is often reduced in acute inflammation (31, 32). Fucosylation of IgG levels was significantly lower in patients than in healthy controls, partly consistent with an earlier study (33). It is known that the absence of fucose increases IgG’s ability to trigger antibody-dependent cytotoxicity (ADCC) by binding to IgG-specific FC-γ receptor IIIa (FcγRIIIa) on natural killer cells (NK), leading to an enhancement of inflammatory cytokines, including interleukin-1β (IL-1β), IL-6, tumor necrosis factor-a (TNF-a) (34, 35). GP4 as an important part of fucosylation can also affect the ADCC pathway (26). Patients with sepsis have inflammation and organ damage. The severity and outcome of the disease are related to the regulation of the balance between these cytokines. To protect damaged organs from further damage, the body has high GP4 expression. The increase in GP4 level is the result of the interaction of inflammatory factor antagonists, which may also be a reasonable explanation for the increase in GP4 in non-surviving patients with sepsis.

Death from sepsis is usually the result of multiple organ dysfunction caused by excessive inflammation (36, 37). In different inflammation-related diseases, the expression level of serum fucosylation, including GP4, is significantly different from that of healthy controls (10, 35, 38). Immunoglobulin G *N*-glycome in patients with infection has attracted worldwide attention due to high mortality and is worthy of further research (26). Therefore, it is necessary to further investigate the relationship between serum immunoglobulin G *N*-glycome level and disease progression in patients with sepsis and its predictive value for hospital death. In our study, we found that the serum immunoglobulin G *N*-glycome concentration of patients with sepsis was significantly different from that of healthy subjects (**Figure 1**), and the serum GP4 concentration of hospitalized patients with sepsis was significantly higher than that of surviving patients. The changes in immunoglobulin G N-glycome concentration may be related to the severity of the disease. Previous studies have shown that SOFA scores play an important role in determining disease severity and prognosis (39, 40). Therefore, we utilized immunoglobulin G *N*-glycome and SOFA scores to assess the risk of death in patients with sepsis. We find that the AUC values of the SOFA score combined with GP4, GP5, and GP9 were 0.76 (95%CI: 0.61 to 0.90), 0.58 (95%CI: 0.40 to 0.7) and 0.57 (95%CI: 0.38 to 0.76). The AUC value of the SOFA score combined with GP4 and GP7 was 0.85 (95%CI: 0.76 to 0.93) in predicting in-hospital mortality in patients with sepsis.

The serum immunoglobulin G *N*-glycome level at admission has predictive value in predicting the risk of hospital death in patients with sepsis. Based on the above advantage, serum immunoglobulin G *N*-glycome may be a valuable predictor of sepsis mortality and is expected to be used in clinical patients with sepsis.

To our knowledge, this study is the first to investigate the clinical value of IgG *N*-glycans in patients with sepsis. However, our study had several limitations. First, the sample size for the IgG *N*-glycan analysis was relatively small. Further large cohort studies are needed to confirm our findings in patients with sepsis. Second, due to the nature of this case-control study, establishing causal relationships between IgG *N*-glycosylation and sepsis is challenging. Third, the study included only Han Chinese populations, so more multicenter studies including patients of different ethnicities are needed to confirm existing data. Fourth, as patients’ conditions and treatment regimens are not exactly the same, it is unclear whether treatment regimens affect the results of this study. In the future, we will explore the relationship between different treatment regimens and serum levels of IgG *N*-glycosylation. Fifth, the study only addressed the *Escherichia coli*-induced sepsis but did not tackle sepsis induced by other pathogens. Sixth, the results of this study can be explained by IgG subtypes as confounders, as those subtypes have not been characterized.

In conclusion, our study found that serum IgG *N*-glycosylation levels at ICU admission were valuable for predicting the in-hospital mortality risk of patients with *Escherichia coli*-induced sepsis, which suggests that IgG *N*-glycosylation may be novel biomarkers that can be used to identify a group of septic patients who have a higher risk of mortality. These findings can be used as a basis for guiding stratified treatment in patients with *Escherichia coli*-induced sepsis.

## Data Availability

The original contributions presented in the study are included in the article/**Supplementary Material**. Further inquiries can be directed to the corresponding authors.

## References

[1] Cohen J Vincent JL Adhikari NK Machado FR Angus DC Calandra T. Sepsis: a roadmap for future research. Lancet Infect Dis. (2015) 15:581–614. doi: 10.1016/S1473-3099(15)70112-X 25932591

[2] Zhou C Liu Y Li Y Shi L. Recent advances and prospects in nanomaterials for bacterial sepsis management. J Mater Chem B. (2023) 11:10778–92. doi: 10.1039/D3TB02220J 37901894

[3] Cao H Gao Y Jia H Zhang L Liu J Mu G. Macrophage-Membrane-Camouflaged nonviral gene vectors for the treatment of multidrug-resistant bacterial sepsis. Nano Lett. (2022) 22:7882–91. doi: 10.1021/acs.nanolett.2c02560 36169350

[4] Fleischmann C Scherag A Adhikari NK Hartog CS Tsaganos T Schlattmann P. Assessment of global incidence and mortality of hospital-treated sepsis. current estimates and limitations. Am J Respir Crit Care Med. (2016) 193:259–72. doi: 10.1164/rccm.201504-0781OC 26414292

[5] Reijnders TDY Saris A Schultz MJ van der Poll T. Immunomodulation by macrolides: therapeutic potential for critical care. Lancet Respir Med. (2020) 8:619–30. doi: 10.1016/S2213-2600(20)30080-1 32526189

[6] Giamarellos-Bourboulis EJ Aschenbrenner AC Bauer M Bock C Calandra T Gat-Viks I. The pathophysiology of sepsis and precision-medicine-based immunotherapy. Nat Immunol. (2024) 25:19–28. doi: 10.1038/s41590-023-01660-5 38168953

[7] Pleass RJ. The therapeutic potential of sialylated Fc domains of human IgG. MAbs. (2021) 13:1953220. doi: 10.1080/19420862.2021.1953220 34288809 PMC8296966

[8] Singh S Thompson JA Yilmaz B Li H Weis S Sobral D. Loss of α-gal during primate evolution enhanced antibody-effector function and resistance to bacterial sepsis. Cell Host Microbe. (2021) 29:347–361.e12. doi: 10.1016/j.chom.2020.12.017 33497603

[9] Wegman AD Waldran MJ Bahr LE Lu JQ Baxter KE Thomas SJ. DENV-specific IgA contributes protective and non-pathologic function during antibody-dependent enhancement of DENV infection. PloS Pathog. (2023) 19:e1011616. doi: 10.1371/journal.ppat.1011616 37639455 PMC10491401

[10] Petrović T Vijay A Vučković F Trbojević-Akmačić I Ollivere BJ Marjanović D. IgG N-glycome changes during the course of severe COVID-19: An observational study. EBioMedicine. (2022) 81:104101. doi: 10.1016/j.ebiom.2022.104101 35773089 PMC9234382

[11] Trzos S Link-Lenczowski P Pocheć E. The role of N-glycosylation in B-cell biology and IgG activity. The aspects of autoimmunity and anti-inflammatory therapy. Front Immunol. (2023) 14:1188838. doi: 10.3389/fimmu.2023.1188838 37575234 PMC10415207

[12] Verhelst X Dias AM Colombel JF Vermeire S Van Vlierberghe H Callewaert N. Protein glycosylation as a diagnostic and prognostic marker of chronic inflammatory gastrointestinal and liver diseases. Gastroenterology. (2020) 158:95–110. doi: 10.1053/j.gastro.2019.08.060 31626754

[13] Cheng HD Tirosh I de Haan N Stöckmann H Adamczyk B McManus CA. IgG Fc glycosylation as an axis of humoral immunity in childhood. J Allergy Clin Immunol. (2020) 145:710–713.e9. doi: 10.1016/j.jaci.2019.10.012 31669096 PMC7010538

[14] Arnold JN Wormald MR Sim RB Rudd PM Dwek RA. The impact of glycosylation on the biological function and structure of human immunoglobulins. Annu Rev Immunol. (2007) 25:21–50. doi: 10.1146/annurev.immunol.25.022106.141702 17029568

[15] Zhang ZJ Wang HF Lian TY Zhou YP Xu XQ Guo F. Human plasma IgG N-Glycome profiles reveal a proinflammatory phenotype in chronic thromboembolic pulmonary hypertension. Hypertension. (2023) 80:1929–39. doi: 10.1161/HYPERTENSIONAHA.123.21408 37449418

[16] García-Alija M van Moer B Sastre DE Azzam T Du JJ Trastoy B. Modulating antibody effector functions by Fc glycoengineering. Biotechnol Adv. (2023) 67:108201. doi: 10.1016/j.biotechadv.2023.108201 37336296 PMC11027751

[17] Shkunnikova S Mijakovac A Sironic L Hanic M Lauc G Kavur MM. IgG glycans in health and disease: Prediction, intervention, prognosis, and therapy. Biotechnol Adv. (2023) 67:108169. doi: 10.1016/j.biotechadv.2023.108169 37207876

[18] World medical association declaration of Helsinki: ethical principles for medical research involving human subjects. Jama. (2013) 310:2191–4. doi: 10.1001/jama.2013.281053 24141714

[19] Singer M Deutschman CS Seymour CW Shankar-Hari M Annane D Bauer M. The third international consensus definitions for sepsis and septic shock (sepsis-3). Jama. (2016) 315:801–10. doi: 10.1001/jama.2016.0287 PMC4968574 26903338

[20] Pucić M Knezević A Vidic J Adamczyk B Novokmet M Polasek O. High throughput isolation and glycosylation analysis of IgG-variability and heritability of the IgG glycome in three isolated human populations. Mol Cell Proteomics. (2011) 10:M111.010090. doi: 10.1074/mcp.M111.010090 PMC3205872 21653738

[21] Liu D Xu X Li Y Zhang J Zhang X Li Q. Immunoglobulin G N-Glycan analysis by ultra-performance liquid chromatography. J Vis Exp. (2020) 155. doi: 10.3791/60104 32009638

[22] Priya S Burns MB Ward T Mars RAT Adamowicz B Lock EF. Identification of shared and disease-specific host gene-microbiome associations across human diseases using multi-omic integration. Nat Microbiol. (2022) 7:780–95. doi: 10.1038/s41564-022-01121-z PMC9159953 35577971

[23] James A Hunter M Straker L Beilby J Bucks R Davis T. Rationale, design and methods for a community-based study of clustering and cumulative effects of chronic disease processes and their effects on ageing: the Busselton healthy ageing study. BMC Public Health. (2013) 13:936. doi: 10.1186/1471-2458-13-936 24099269 PMC3852572

[24] Li X Wang H Russell A Cao W Wang X Ge S. Type 2 Diabetes Mellitus is associated with the immunoglobulin G N-Glycome through putative proinflammatory mechanisms in an Australian Population. Omics. (2019) 23:631–9. doi: 10.1089/omi.2019.0075 31526239

[25] Saxena J Das S Kumar A Sharma A Sharma L Kaushik S. Biomarkers in sepsis. Clinica Chimica Acta; Int J Clin Chem. (2024) 562:119891. doi: 10.1016/j.cca.2024.119891 39067500

[26] Cindrić A Pribić T Lauc G. High-throughput N-glycan analysis in aging and inflammaging: State of the art and future directions. Semin In Immunol. (2024) 73:101890. doi: 10.1016/j.smim.2024.101890 39383621

[27] Cajander S Kox M Scicluna BP Weigand MA Mora RA Flohé SB. Profiling the dysregulated immune response in sepsis: overcoming challenges to achieve the goal of precision medicine. Lancet Respir Med. (2024) 12:305–22. doi: 10.1016/S2213-2600(23)00330-2 38142698

[28] Vicente MM Alves I Gaifem J Rodrigues CS Fernandes Â Dias AM. Altered IgG glycosylation at COVID-19 diagnosis predicts disease severity. Eur J Immunol. (2022) 52:946–57. doi: 10.1002/eji.202149491 PMC9087392 35307819

[29] Haslund-Gourley BS Wigdahl B Comunale MA. IgG N-glycan signatures as potential diagnostic and prognostic biomarkers. Diagn (Basel). (2023) 13. doi: 10.3390/diagnostics13061016 PMC10047871 36980324

[30] Klarić L Tsepilov YA Stanton CM Mangino M Sikka TT Esko T. Glycosylation of immunoglobulin G is regulated by a large network of genes pleiotropic with inflammatory diseases. Sci Adv. (2020) 6:eaax0301. doi: 10.1126/sciadv.aax0301 32128391 PMC7030929

[31] Kljaković-Gašpić-Batinjan M Petrović T Vučković F Hadžibegović I Radovani B Jurin I. Differences in Immunoglobulin G glycosylation between influenza and COVID-19 patients. Engineering. (2022) 26:54–62. doi: 10.1016/j.eng.2022.08.007 PMC9446557 36093331

[32] Seeling M Brückner C Nimmerjahn F. Differential antibody glycosylation in autoimmunity: sweet biomarker or modulator of disease activity? Nat Rev Rheumatol. (2017) 13:621–30. doi: 10.1038/nrrheum.2017.146 28905852

[33] Lu X Wang L Wang M Li Y Zhao Q Shi Y. Association between immunoglobulin G N-glycosylation and lupus nephritis in female patients with systemic lupus erythematosus: a case-control study. Front In Immunol. (2023) 14:1257906. doi: 10.3389/fimmu.2023.1257906 37809087 PMC10552529

[34] Chakraborty S Gonzalez J Edwards K Mallajosyula V Buzzanco AS Sherwood R. Proinflammatory IgG Fc structures in patients with severe COVID-19. Nat Immunol. (2021) 22:67–73. doi: 10.1038/s41590-020-00828-7 33169014 PMC8130642

[35] Zhang Z-J Liu C Ma JL Ma JS Wang J Li RN. Prognostic value of plasma immunoglobulin G N-Glycome traits in pulmonary arterial hypertension. J Am Coll Cardiol. (2024) 84:1092–103. doi: 10.1016/j.jacc.2024.05.077 39260931

[36] De Backer D Deutschman CS Hellman J Myatra SN Ostermann M Prescott HC. Surviving sepsis campaign research priorities 2023. Crit Care Med. (2024) 52:268–96. doi: 10.1097/CCM.0000000000006135 38240508

[37] Evrard B Sinha P Delucchi K Hendrickson CM Kangelaris KN Liu KD. Causes and attributable fraction of death from ARDS in inflammatory phenotypes of sepsis. Crit Care (London England). (2024) 28:164. doi: 10.1186/s13054-024-04943-x PMC11092165 38745253

[38] Hanić M Vučković F Deriš H Bewshea C Lin S Goodhand JR. Anti-TNF biologicals enhance the anti-inflammatory properties of IgG N-Glycome in Crohn’s Disease. Biomolecules. (2023) 13. doi: 10.3390/biom13060954 PMC10295852 37371534

[39] Wang H Wang M Chen J Hou H Guo Z Yang H. Interleukin-36 is overexpressed in human sepsis and IL-36 receptor deletion aggravates lung injury and mortality through epithelial cells and fibroblasts in experimental murine sepsis. Crit Care (London England). (2023) 27:490. doi: 10.1186/s13054-023-04777-z PMC10717293 38093296

[40] Lan L Zhou M Chen X Dai M Wang L Li H. Prognostic accuracy of SOFA, MEWS, and SIRS criteria in predicting the mortality rate of patients with sepsis: A Meta-analysis. Nurs In Crit Care. (2023) 29:1623–35. doi: 10.1111/nicc.13016 38129945

