# Supplementary material for: Immunoglobulin G N-Glycome as a biomarker of mortality risk in Escherichia coli induced sepsis
Source: Front Immunol. 2025 Mar 17;16:1532145. doi: 10.3389/fimmu.2025.1532145 (PMC11955649; doi:10.3389/fimmu.2025.1532145)
Supplement: Supplementary file 1 [file Table1.docx]

[Supplement]

Table S1 IgG *N*-glycan traits between controls and patients with sepsis

| Initially glycans | Controls (n=100) | Sepsis (n=100) | *Z* | *P* |
| --- | --- | --- | --- | --- |
| GP1 | 0.20 (0.10, 0.35) | 0.36 (0.24, 0.54) | -5.72 | 1.05E-08 |
| GP2 | 0.39 (0.25, 0.59) | 0.83 (0.52, 1.36) | -6.93 | 4.10E-12 |
| GP3 | 0.34 (0.22, 0.46) | 0.19 (0.13, 0.31) | -4.71 | 2.46E-06 |
| GP4 | 22.51 (18.13, 25.09) | 27.42 (22.67, 32.66) | -6.00 | 1.93E-09 |
| GP5 | 0.35 (0.15, 0.49) | 0.10 (0.08, 0.14) | -7.12 | 1.08E-12 |
| GP6 | 4.67 (3.83, 5.78) | 5.66 (4.58, 6.53) | -4.64 | 3.55E-06 |
| GP7 | 0.39 (0.24, 0.59) | 0.45 (0.31, 0.57) | -1.61 | 0.11 |
| GP8 | 18.70 (17.37, 19.93) | 17.40 (16.20, 18.19) | -4.91 | 8.88E-07 |
| GP9 | 9.73 (8.51, 10.44) | 9.25 (7.97, 10.07) | -1.96 | 0.05 |
| GP10 | 4.04 (3.49, 4.59) | 4.29 (3.76, 4.80) | -1.96 | 0.05 |
| GP11 | 1.03 (0.75, 1.40) | 0.79 (0.69, 0.92) | -4.51 | 6.37E-06 |
| GP12 | 0.61 (0.39, 0.90) | 0.54 (0.38, 0.82) | -1.10 | 0.27 |
| GP13 | 0.45 (0.31, 0.62) | 0.29 (0.23, 0.35) | -5.87 | 4.26E-09 |
| GP14 | 14.32 (12.29, 17.21) | 9.96 (7.71, 12.04) | -8.50 | 1.95E-17 |
| GP15 | 1.23 (1.04, 1.55) | 1.42 (1.31, 1.62) | -3.76 | 1.70E-04 |
| GP16 | 3.01 (2.70, 3.41) | 3.28 (2.68, 3.62) | -1.97 | 0.05 |
| GP17 | 0.74 (0.63, 0.89) | 0.97 (0.84, 1.08) | -7.24 | 4.54E-13 |
| GP18 | 9.96 (8.04, 12.51) | 7.57 (6.08, 9.19) | -6.02 | 1.76E-09 |
| GP19 | 1.85 (1.64, 2.14) | 2.21 (1.98, 2.50) | -5.51 | 3.63E-08 |
| GP20 | 0.11 (0.07, 0.16) | 0.16 (0.13, 0.21) | -5.86 | 4.59E-09 |
| GP21 | 0.60 (0.41, 0.72) | 0.83 (0.73, 0.97) | -8.43 | 3.42E-17 |
| GP22 | 0.18 (0.13, 0.25) | 0.23 (0.18, 0.28) | -3.69 | 2.27E-04 |
| GP23 | 1.76 (1.45, 2.03) | 1.92 (1.69, 2.43) | -2.86 | 4.23E-03 |
| GP24 | 2.16 (1.74, 2.41) | 2.53 (2.14, 3.08) | -5.29 | 1.20E-07 |

GP, glycan peak; Mann-Whitney U Test was used.

Table S2 The IgG deprived *N*-glycan

| Deprived glycans | Controls (n=100) | Sepsis (n=100) | *Z* | *P* |
| --- | --- | --- | --- | --- |
| FGS/(FG+FGS) | 25.23 (23.08, 28.40) | 26.06 (23.90, 28.51) | -0.98 | 0.33 |
| FBGS/(FBG+FBGS) | 38.26 (34.98, 43.28) | 42.98 (39.49, 46.10) | -4.02 | 5.75E-05 |
| FGS/(F+FG+FGS) | 18.34 (15.71, 22.40) | 16.67 (14.25, 18.75) | -3.31 | 9.38E-04 |
| FBGS/(FB+FBG+FBGS) | 26.20 (23.11, 30.30) | 28.35 (25.17, 31.63) | -2.26 | 0.02 |
| FG1S1/(FG1+FG1S1) | 9.66 (8.61, 10.98) | 10.79 (9.64, 12.06) | -4.04 | 5.31E-05 |
| FG2S1/(FG2+FG2S1+FG2S2) | 38.04 (35.94, 40.02) | 38.97 (37.50, 41.23) | -3.50 | 4.67E-04 |
| FG2S2/(FG2+FG2S1+FG2S2) | 6.65 (5.56, 8.07) | 10.02 (8.94, 12.26) | -9.24 | 2.50E-20 |
| FBG2S1/(FBG2+FBG2S1+FBG2S2) | 35.39 (31.91, 39.48) | 35.73 (32.94, 38.13) | -0.22 | 0.83 |
| FBG2S2/(FBG2+FBG2S1+FBG2S2) | 40.63 (35.66, 43.75) | 40.99 (37.84, 43.63) | -0.92 | 0.36 |
| F^total^S1/F^total^S2 | 3.87 (3.29, 4.45) | 2.84 (2.58, 3.25) | -8.67 | 4.45E-18 |
| FS1/FS2 | 7.39 (6.21, 8.66) | 5.44 (4.82, 6.04) | -7.86 | 3.98E-15 |
| FBS1/FBS2 | 0.89 (0.75, 1.07) | 0.88 (0.79, 0.98) | -0.68 | 0.50 |
| FBS^total^/FS^total^ | 0.26 (0.23, 0.34) | 0.37 (0.33, 0.45) | -7.33 | 2.30E-13 |
| FBS1/FS1 | 0.14 (0.12, 0.18) | 0.20 (0.18, 0.25) | -7.05 | 1.80E-12 |
| FBS1/(FS1+FBS1) | 0.12 (0.10, 0.15) | 0.17 (0.15, 0.20) | -7.05 | 1.80E-12 |
| FBS2/FS2 | 1.21 (1.00, 1.43) | 1.28 (1.08, 1.52) | -1.85 | 0.06 |
| FBS2/(FS2+FBS2) | 0.55 (0.50, 0.59) | 0.56 (0.52, 0.60) | -1.85 | 0.06 |
| GP1^n^ | 0.26 (0.14, 0.42) | 0.44 (0.30, 0.65) | -5.82 | 6.05E-09 |
| GP2^n^ | 0.49 (0.33, 0.74) | 1.06 (0.65, 1.62) | -7.00 | 2.51E-12 |
| GP4^n^ | 28.21 (22.87, 31.09) | 34.27 (29.22, 40.08) | -6.44 | 1.17E-10 |
| GP5^n^ | 0.43 (0.20, 0.61) | 0.13 (0.10, 0.17) | -7.20 | 6.10E-13 |
| GP6^n^ | 5.93 (4.95, 7.02) | 7.11 (5.92, 7.97) | -4.88 | 1.08E-06 |
| GP7^n^ | 0.49 (0.31, 0.76) | 0.55 (0.40, 0.75) | -1.59 | 0.11 |
| GP8^n^ | 23.71 (21.59, 25.09) | 21.77 (20.26, 23.41) | -4.71 | 2.47E-06 |
| GP9^n^ | 12.29 (10.77, 13.26) | 11.79 (9.89, 13.14) | -1.69 | 0.09 |
| GP10^n^ | 5.13 (4.41, 5.83) | 5.48 (4.67, 6.10) | -1.91 | 0.06 |
| GP11^n^ | 1.32 (0.92, 1.76) | 0.98 (0.86, 1.14) | -4.76 | 1.92E-06 |
| GP12^n^ | 0.77 (0.48, 1.16) | 0.68 (0.47, 1.03) | -1.17 | 0.24 |
| GP13^n^ | 0.55 (0.39, 0.79) | 0.36 (0.29, 0.45) | -5.75 | 8.71E-09 |
| GP14^n^ | 18.18 (14.99, 22.30) | 12.29 (9.53, 15.38) | -7.87 | 3.54E-15 |
| GP15^n^ | 1.53 (1.28, 1.97) | 1.77 (1.61, 2.04) | -3.52 | 4.38E-04 |
| F^n total^ | 96.43 (95.70, 97.46) | 96.71 (95.69, 97.57) | -0.62 | 0.54 |
| FG0^n total^/G0^n^ | 98.54 (97.91, 98.93) | 97.48 (96.34, 98.46) | -5.54 | 3.00E-08 |
| FG1^n total^/G1^n^ | 98.87 (98.28, 99.30) | 98.62 (98.16, 99.02) | -2.63 | 0.01 |
| FG2^n total^ /G2^n^ | 93.48 (91.88, 94.97) | 92.58 (90.91, 94.36) | -2.67 | 0.01 |
| F^n^ | 82.24 (79.98, 84.35) | 81.92 (79.55, 83.30) | -1.96 | 0.05 |
| FG0^n^/G0^n^ | 80.48 (78.08, 83.09) | 81.59 (77.82, 83.32) | -0.85 | 0.39 |
| FG1^n^/G1^n^ | 83.91 (81.77, 85.70) | 82.84 (80.56, 84.53) | -2.63 | 0.01 |
| FG2^n^/G2^n^ | 85.75 (83.43, 87.55) | 80.59 (77.18, 82.62) | -9.11 | 8.32E-20 |
| FB^n^ | 13.91 (12.48, 15.97) | 14.98 (13.54, 17.02) | -3.06 | 2.24E-03 |
| FBG0^n^/G0^n^ | 17.89 (15.68, 20.00) | 16.16 (13.94, 19.16) | -2.83 | 4.59E-03 |
| FBG1^n^/G1^n^ | 14.94 (13.21, 16.84) | 15.59 (14.18, 17.86) | -2.37 | 0.02 |
| FBG2^n^/G2^n^ | 7.75 (6.56, 8.83) | 12.12 (10.56, 13.85) | -10.58 | 3.69E-26 |
| FB^n^/F^n^ | 16.90 (15.11, 19.96) | 18.58 (16.22, 21.30) | -2.94 | 3.26E-03 |
| FB^n^/F^n total^ | 14.46 (13.12, 16.64) | 15.67 (13.96, 17.56) | -2.94 | 3.26E-03 |
| F^n^/(B^n^ + FB^n^) | 5.65 (4.82, 6.34) | 5.23 (4.56, 6.01) | -2.32 | 0.02 |
| B^n^/(F^n^ + FB^n^) | 5.73 (3.99, 8.23) | 3.71 (2.98, 4.73) | -5.65 | 1.57E-08 |
| FBG2^n^/FG2^n^ | 0.09 (0.08, 0.11) | 0.15 (0.13, 0.18) | -10.63 | 2.08E-26 |
| FBG2^n^ /(FG2^n^ + FBG2^n^ ) | 8.36 (7.02, 9.56) | 13.16 (11.28, 15.33) | -10.63 | 2.08E-26 |
| FG2^n^/(BG2^n^ + FBG2^n^) | 8.10 (6.83, 9.29) | 5.50 (4.64, 6.43) | -9.66 | 4.62E-22 |
| BG2^n^/(FG2^n^ + FBG2^n^) | 28.24 (19.80, 38.62) | 26.03 (20.17, 33.78) | -0.84 | 0.40 |

Mann-Whitney U Test was used.

Table S3 IgG *N*-glycan traits in septic survivor and septic non-survivor during hospitalization

| Glycans | Septic survivor (n=84) | Septic non-survivor (n=16) | *Z* | *P* |
| --- | --- | --- | --- | --- |
| GP1 | 0.37 (0.25, 0.54) | 0.31 (0.22, 0.51) | -0.63 | 0.53 |
| GP2 | 0.87 (0.54, 1.42) | 0.74 (0.45, 1.09) | -1.17 | 0.24 |
| GP3 | 0.20 (0.14, 0.32) | 0.18 (0.12, 0.26) | -0.72 | 0.47 |
| GP4 | 27.16 (22.12, 30.82) | 33.52 (26.25, 38.72) | -2.99 | 2.83E-03 |
| GP5 | 0.10 (0.08, 0.14) | 0.13 (0.06, 0.16) | -0.64 | 0.52 |
| GP6 | 5.66 (4.58, 6.53) | 5.68 (4.54, 6.62) | -0.20 | 0.84 |
| GP7 | 0.48 (0.33, 0.60) | 0.32 (0.24, 0.38) | -3.48 | 5.02E-04 |
| GP8 | 17.52 (16.36, 18.26) | 16.82 (15.04, 17.41) | -1.95 | 0.05 |
| GP9 | 9.26 (8.06, 9.92) | 9.24 (7.54, 10.75) | -0.07 | 0.95 |
| GP10 | 4.34 (3.84, 4.88) | 4.08 (2.93, 4.48) | -2.28 | 0.02 |
| GP11 | 0.79 (0.69, 0.92) | 0.76 (0.66, 0.91) | -0.64 | 0.52 |
| GP12 | 0.62 (0.40, 0.86) | 0.35 (0.29, 0.47) | -3.66 | 2.49E-04 |
| GP13 | 0.30 (0.25, 0.36) | 0.19 (0.16, 0.26) | -4.23 | 2.33E-05 |
| GP14 | 10.27 (8.03, 12.39) | 7.56 (6.18, 10.73) | -2.57 | 0.01 |
| GP15 | 1.44 (1.34, 1.65) | 1.35 (1.07, 1.47) | -2.51 | 0.01 |
| GP16 | 3.28 (2.64, 3.68) | 3.23 (2.74, 3.53) | -0.39 | 0.70 |
| GP17 | 0.97 (0.85, 1.08) | 0.95 (0.83, 1.07) | -0.71 | 0.48 |
| GP18 | 7.93 (6.39, 9.21) | 6.52 (4.88, 7.99) | -2.28 | 0.02 |
| GP19 | 2.21 (1.99, 2.53) | 2.23 (1.77, 2.46) | -0.45 | 0.66 |
| GP20 | 0.17 (0.14, 0.21) | 0.15 (0.12, 0.22) | -0.78 | 0.44 |
| GP21 | 0.84 (0.76, 0.97) | 0.72 (0.64, 0.89) | -2.59 | 0.01 |
| GP22 | 0.24 (0.19, 0.29) | 0.19 (0.15, 0.24) | -2.42 | 0.02 |
| GP23 | 1.93 (1.69, 2.43) | 1.86 (1.66, 2.42) | -0.24 | 0.81 |
| GP24 | 2.52 (2.14, 3.12) | 2.62 (2.10, 3.00) | -0.17 | 0.86 |
| FGS/(FG+FGS) | 26.16 (23.90, 28.64) | 24.84 (23.84, 27.30) | -0.60 | 0.55 |
| FBGS/(FBG+FBGS) | 42.24 (39.17, 45.61) | 46.10 (40.88, 47.05) | -2.25 | 0.02 |
| FGS/(F+FG+FGS) | 16.84 (14.85, 18.92) | 14.56 (12.12, 17.87) | -2.34 | 0.02 |
| FBGS/(FB+FBG+FBGS) | 28.21 (25.13, 31.43) | 29.58 (25.42, 32.33) | -0.79 | 0.43 |
| FG1S1/(FG1+FG1S1) | 10.79 (9.62, 12.06) | 10.88 (9.89, 11.98) | -0.31 | 0.76 |
| FG2S1/(FG2+FG2S1+FG2S2) | 38.95 (37.62, 40.92) | 39.25 (37.12, 41.82) | -0.41 | 0.68 |
| FG2S2/(FG2+FG2S1+FG2S2) | 9.76 (8.61, 11.78) | 12.13 (10.46, 14.45) | -3.05 | 2.32E-03 |
| FBG2S1/(FBG2+FBG2S1+FBG2S2) | 35.73 (32.85, 38.13) | 36.36 (34.07, 39.67) | -0.81 | 0.42 |
| FBG2S2/(FBG2+FBG2S1+FBG2S2) | 40.78 (37.75, 43.44) | 41.88 (38.33, 45.76) | -1.36 | 0.17 |
| F^total^S1/F^total^S2 | 2.89 (2.63, 3.26) | 2.57 (2.30, 2.98) | -2.55 | 0.01 |
| FS1/FS2 | 5.53 (4.96, 6.21) | 5.04 (4.27, 5.62) | -2.29 | 0.02 |
| FBS1/FBS2 | 0.88 (0.79, 0.97) | 0.86 (0.76, 1.01) | -0.27 | 0.79 |
| FBS^total^/FS^total^ | 0.37 (0.32, 0.43) | 0.42 (0.34, 0.47) | -1.49 | 0.14 |
| FBS1/FS1 | 0.20 (0.17, 0.25) | 0.22 (0.18, 0.26) | -1.36 | 0.17 |
| FBS1/(FS1+FBS1) | 0.16 (0.15, 0.20) | 0.18 (0.16, 0.21) | -1.36 | 0.17 |
| FBS2/FS2 | 1.27 (1.08, 1.52) | 1.32 (1.09, 1.57) | -0.02 | 0.98 |
| FBS2/(FS2+FBS2) | 0.56 (0.52, 0.60) | 0.57 (0.52, 0.61) | -0.02 | 0.98 |
| GP1^n^ | 0.45 (0.31, 0.67) | 0.40 (0.28, 0.59) | -0.87 | 0.39 |
| GP2^n^ | 1.10 (0.68, 1.75) | 0.94 (0.52, 1.30) | -1.49 | 0.14 |
| GP4^n^ | 34.07 (28.66, 38.13) | 42.02 (33.88, 46.36) | -3.12 | 1.80E-03 |
| GP5^n^ | 0.13 (0.10, 0.17) | 0.15 (0.08, 0.21) | -0.45 | 0.65 |
| GP6^n^ | 7.11 (5.95, 7.97) | 7.16 (5.33, 7.96) | -0.77 | 0.44 |
| GP7^n^ | 0.59 (0.41, 0.78) | 0.38 (0.30, 0.47) | -3.67 | 2.46E-04 |
| GP8^n^ | 22.08 (20.56, 23.64) | 21.07 (17.94, 21.94) | -2.11 | 0.04 |
| GP9^n^ | 11.79 (10.04, 13.05) | 11.68 (9.20, 13.55) | -0.15 | 0.88 |
| GP10^n^ | 5.52 (4.77, 6.32) | 4.97 (3.44, 5.52) | -2.61 | 0.01 |
| GP11^n^ | 0.99 (0.88, 1.14) | 0.93 (0.79, 1.18) | -1.05 | 0.29 |
| GP12^n^ | 0.79 (0.51, 1.09) | 0.43 (0.37, 0.58) | -3.70 | 2.20E-04 |
| GP13^n^ | 0.37 (0.32, 0.47) | 0.24 (0.20, 0.33) | -4.28 | 1.89E-05 |
| GP14^n^ | 12.99 (9.91, 15.47) | 9.38 (7.41, 13.90) | -2.55 | 0.01 |
| GP15^n^ | 1.80 (1.61, 2.17) | 1.69 (1.28, 1.82) | -2.42 | 0.02 |
| F^n total^ | 96.50 (95.59, 97.40) | 97.59 (96.91, 97.94) | -3.32 | 9.03E-04 |
| FG0^n total^/G0^n^ | 97.32 (95.98, 98.43) | 98.10 (97.62, 98.73) | -2.44 | 0.01 |
| FG1^n total^/G1^n^ | 98.49 (98.06, 98.99) | 99.00 (98.68, 99.16) | -2.93 | 3.35E-03 |
| FG2^n total^ /G2^n^ | 92.19 (90.06, 94.34) | 93.94 (92.71, 94.79) | -2.22 | 0.03 |
| F^n^ | 81.08 (79.30, 82.89) | 83.02 (81.85, 86.15) | -3.02 | 2.54E-03 |
| FG0^n^/G0^n^ | 81.13 (77.39, 82.89) | 83.45 (82.11, 87.14) | -3.64 | 2.74E-04 |
| FG1^n^/G1^n^ | 82.48 (79.58, 84.49) | 83.63 (82.31, 86.31) | -1.95 | 0.05 |
| FG2^n^/G2^n^ | 80.38 (77.18, 82.62) | 80.97 (76.17, 82.85) | -0.26 | 0.80 |
| FB^n^ | 15.09 (13.60, 17.31) | 14.28 (11.13, 15.51) | -1.96 | 0.05 |
| FBG0^n^/G0^n^ | 16.44 (14.62, 19.49) | 14.76 (11.36, 15.69) | -3.25 | 1.14E-03 |
| FBG1^n^/G1^n^ | 15.87 (14.20, 18.23) | 15.18 (12.68, 16.52) | -1.45 | 0.15 |
| FBG2^n^/G2^n^ | 11.64 (10.46, 13.66) | 13.35 (12.38, 15.55) | -2.33 | 0.02 |
| FB^n^/F^n^ | 18.79 (16.34, 22.29) | 17.11 (12.92, 19.01) | -2.14 | 0.03 |
| FB^n^/F^n total^ | 15.82 (14.05, 18.23) | 14.61 (11.43, 15.97) | -2.14 | 0.03 |
| F^n^/(B^n^ + FB^n^) | 5.17 (4.35, 5.97) | 5.77 (5.15, 7.51) | -2.37 | 0.02 |
| B^n^/(F^n^ + FB^n^) | 3.91 (3.30, 4.81) | 2.44 (2.08, 3.40) | -4.29 | 1.81E-05 |
| FBG2^n^/FG2^n^ | 0.14 (0.13, 0.18) | 0.17 (0.15, 0.20) | -1.85 | 0.06 |
| FBG2^n^ /(FG2^n^ + FBG2^n^ ) | 12.57 (11.23, 15.22) | 14.22 (13.15, 16.96) | -1.85 | 0.06 |
| FG2^n^/(BG2^n^ + FBG2^n^) | 5.55 (4.71, 6.50) | 5.23 (4.07, 5.75) | -1.17 | 0.24 |
| BG2^n^/(FG2^n^ + FBG2^n^) | 26.25 (20.84, 34.17) | 23.51 (18.35, 31.81) | -1.10 | 0.27 |
| Fucosylation | 94.97 (94.11, 95.87) | 96.02 (95.46, 96.44) | -3.22 | 1.30E-03 |
| Bisecting GlcNAc | 17.38 (15.99, 20.01) | 17.16 (14.10, 18.59) | -1.57 | 0.12 |
| Sialylation | 20.10 (18.12, 22.62) | 17.88 (15.74, 21.60) | -1.96 | 0.05 |
| Galactosylation |  |  |  |  |
| G0 | 34.58 (28.71, 38.95) | 40.97 (32.63, 45.37) | -2.53 | 0.01 |
| G1 | 32.66 (31.17, 33.72) | 30.63 (29.21, 32.55) | -2.26 | 0.02 |
| G2 | 12.98 (10.28, 14.67) | 9.36 (7.84, 12.96) | -2.90 | 3.78E-03 |

GP, glycan peak; G0, agalactosylation; G1, monogalactosylation; G2, digalactosylation; Mann-Whitney U Test was used.

Table S4 Subgroup analysis of IgG *N*-glycan traits on gender in the septic patients

| Glycans | Male (n=52) | Female (n=48) | Z | *P* |
| --- | --- | --- | --- | --- |
| GP1 | 0.26 (0.37, 0.53) | 0.21 (0.33, 0.55) | -1.10 | 0.27 |
| GP2 | 0.57 (0.88, 1.41) | 0.5 (0.76, 1.24) | -0.99 | 0.32 |
| GP3 | 0.14 (0.21, 0.35) | 0.12 (0.19, 0.28) | -1.31 | 0.19 |
| GP4 | 22.67 (28.26, 33.23) | 22.48 (27.16, 30.78) | -0.99 | 0.32 |
| GP5 | 0.08 (0.11, 0.15) | 0.07 (0.1, 0.14) | -0.91 | 0.36 |
| GP6 | 4.53 (5.6, 6.32) | 4.65 (5.9, 6.99) | -1.17 | 0.24 |
| GP7 | 0.31 (0.47, 0.59) | 0.31 (0.41, 0.57) | -0.52 | 0.60 |
| GP8 | 16.1 (17.37, 17.99) | 16.2 (17.42, 18.74) | -0.73 | 0.46 |
| GP9 | 7.81 (9.2, 10.34) | 8.27 (9.27, 9.97) | -0.33 | 0.74 |
| GP10 | 3.39 (4.27, 4.79) | 3.88 (4.36, 4.99) | -1.19 | 0.23 |
| GP11 | 0.68 (0.77, 0.89) | 0.69 (0.8, 0.94) | -0.64 | 0.52 |
| GP12 | 0.36 (0.47, 0.81) | 0.42 (0.6, 0.83) | -1.00 | 0.32 |
| GP13 | 0.23 (0.28, 0.37) | 0.24 (0.3, 0.35) | -0.27 | 0.79 |
| GP14 | 7.42 (9.34, 11.82) | 8.2 (10.15, 12.42) | -1.09 | 0.28 |
| GP15 | 1.26 (1.39, 1.61) | 1.36 (1.44, 1.65) | -1.55 | 0.12 |
| GP16 | 2.78 (3.3, 3.57) | 2.63 (3.24, 3.68) | -0.22 | 0.82 |
| GP17 | 0.86 (0.96, 1.07) | 0.82 (0.97, 1.1) | -0.09 | 0.93 |
| GP18 | 5.93 (7.12, 8.8) | 6.37 (7.93, 9.21) | -0.64 | 0.52 |
| GP19 | 1.92 (2.17, 2.42) | 2 (2.24, 2.65) | -1.49 | 0.14 |
| GP20 | 0.13 (0.16, 0.21) | 0.13 (0.17, 0.22) | -0.13 | 0.90 |
| GP21 | 0.7 (0.82, 0.99) | 0.75 (0.83, 0.96) | -0.38 | 0.70 |
| GP22 | 0.17 (0.23, 0.28) | 0.18 (0.22, 0.28) | -0.24 | 0.81 |
| GP23 | 1.73 (2.05, 2.49) | 1.59 (1.83, 2.22) | -1.40 | 0.16 |
| GP24 | 2.14 (2.48, 3.03) | 2.12 (2.56, 3.12) | -0.59 | 0.56 |
| FGS/(FG+FGS) | 24.14 (26.22, 28.67) | 23.61 (25.8, 28.3) | -0.92 | 0.36 |
| FBGS/(FBG+FBGS) | 39.63 (43.77, 46.08) | 39.17 (41.88, 46.25) | -0.76 | 0.45 |
| FGS/(F+FG+FGS) | 14.18 (16.25, 18.73) | 14.92 (16.73, 18.85) | -0.41 | 0.68 |
| FBGS/(FB+FBG+FBGS) | 25.1 (28.9, 32.01) | 25.48 (27.75, 31.55) | -0.47 | 0.64 |
| FG1S1/(FG1+FG1S1) | 9.78 (10.95, 11.82) | 9.41 (10.65, 12.16) | -0.92 | 0.36 |
| FG2S1/(FG2+FG2S1+FG2S2) | 37.5 (39.08, 41.72) | 37.4 (38.95, 40.86) | -0.97 | 0.33 |
| FG2S2/(FG2+FG2S1+FG2S2) | 9.19 (10.97, 12.44) | 8.5 (9.63, 11.22) | -2.08 | 0.04 |
| FBG2S1/(FBG2+FBG2S1+FBG2S2) | 32.85 (35.5, 37.24) | 33.13 (36.08, 38.65) | -1.01 | 0.31 |
| FBG2S2/(FBG2+FBG2S1+FBG2S2) | 37.75 (41.62, 44.35) | 38.04 (40.38, 43.33) | -1.28 | 0.20 |
| F^total^S1/F^total^S2 | 2.5 (2.82, 3.13) | 2.63 (2.92, 3.26) | -1.03 | 0.30 |
| FS1/FS2 | 4.63 (5.39, 5.85) | 5.11 (5.62, 6.33) | -1.74 | 0.08 |
| FBS1/FBS2 | 0.76 (0.86, 0.96) | 0.8 (0.9, 0.98) | -1.23 | 0.22 |
| FBS^total^/FS^total^ | 0.33 (0.36, 0.43) | 0.31 (0.38, 0.46) | -0.68 | 0.49 |
| FBS1/FS1 | 0.18 (0.2, 0.25) | 0.17 (0.21, 0.27) | -0.63 | 0.53 |
| FBS1/(FS1+FBS1) | 0.15 (0.16, 0.2) | 0.15 (0.17, 0.21) | -0.63 | 0.53 |
| FBS2/FS2 | 1.03 (1.24, 1.46) | 1.1 (1.4, 1.6) | -1.42 | 0.16 |
| FBS2/(FS2+FBS2) | 0.51 (0.55, 0.59) | 0.52 (0.58, 0.62) | -1.42 | 0.16 |
| GP1^n^ | 0.35 (0.46, 0.64) | 0.28 (0.42, 0.68) | -1.07 | 0.28 |
| GP2^n^ | 0.7 (1.1, 1.78) | 0.63 (1, 1.54) | -1.01 | 0.31 |
| GP4^n^ | 29.47 (34.82, 40.98) | 29.05 (33.85, 38.25) | -1.00 | 0.32 |
| GP5^n^ | 0.1 (0.13, 0.18) | 0.09 (0.12, 0.17) | -0.83 | 0.40 |
| GP6^n^ | 5.8 (7.05, 7.75) | 5.96 (7.45, 8.77) | -1.24 | 0.21 |
| GP7^n^ | 0.37 (0.58, 0.76) | 0.4 (0.52, 0.74) | -0.50 | 0.62 |
| GP8^n^ | 19.84 (21.63, 23.17) | 20.64 (22.08, 23.55) | -0.71 | 0.48 |
| GP9^n^ | 9.44 (11.74, 13.18) | 10.04 (11.81, 12.79) | -0.26 | 0.80 |
| GP10^n^ | 4.4 (5.4, 6.09) | 4.85 (5.48, 6.27) | -1.05 | 0.29 |
| GP11^n^ | 0.86 (0.98, 1.12) | 0.86 (1, 1.17) | -0.53 | 0.60 |
| GP12^n^ | 0.45 (0.59, 1.03) | 0.51 (0.77, 1.04) | -0.90 | 0.37 |
| GP13^n^ | 0.29 (0.35, 0.47) | 0.31 (0.37, 0.44) | -0.28 | 0.78 |
| GP14^n^ | 9.06 (11.38, 15.06) | 10.12 (12.95, 15.61) | -1.06 | 0.29 |
| GP15^n^ | 1.56 (1.72, 2.03) | 1.65 (1.8, 2.15) | -1.24 | 0.21 |
| GPn | 77.48 (80.37, 82.16) | 77.02 (79.62, 81.88) | -0.38 | 0.70 |
| G0^n^ | 37.97 (44.38, 50.8) | 35.76 (42.64, 48.9) | -0.89 | 0.37 |
| G1^n^ | 37.31 (40.75, 42.51) | 38.93 (41.05, 43.21) | -0.84 | 0.40 |
| G2^n^ | 11.68 (14, 18.34) | 12.74 (16.1, 18.39) | -0.98 | 0.33 |
| F^n total^ | 95.66 (96.57, 97.62) | 95.84 (97.06, 97.57) | -0.77 | 0.44 |
| FG0^n total^/G0^n^ | 96.17 (97.35, 98.33) | 96.46 (97.78, 98.52) | -0.86 | 0.39 |
| FG1^n total^/G1^n^ | 98.13 (98.5, 99.03) | 98.2 (98.7, 99.02) | -0.80 | 0.42 |
| FG2^n total^ /G2^n^ | 90.75 (92.6, 94.29) | 90.95 (92.51, 94.5) | -0.23 | 0.81 |
| F^n^ | 80.2 (82.14, 83.37) | 77.9 (81.23, 83.26) | -0.81 | 0.42 |
| FG0^n^/G0^n^ | 78.24 (82.34, 83.37) | 76.6 (81.17, 83.1) | -1.15 | 0.25 |
| FG1^n^/G1^n^ | 81.57 (83.15, 84.47) | 79.47 (82.31, 85.05) | -0.86 | 0.39 |
| FG2^n^/G2^n^ | 76.8 (80.19, 82.52) | 78.25 (80.79, 82.77) | -0.42 | 0.67 |
| FB^n^ | 13.58 (14.7, 16.02) | 13.43 (15.44, 17.97) | -1.23 | 0.22 |
| FBG0^n^/G0^n^ | 13.74 (15.76, 17.46) | 14.9 (16.53, 19.49) | -1.45 | 0.15 |
| FBG1^n^/G1^n^ | 14.2 (15.24, 17.27) | 13.99 (16.36, 18.6) | -1.04 | 0.30 |
| FBG2^n^/G2^n^ | 10.68 (12.14, 13.99) | 10.46 (11.54, 13.77) | -0.69 | 0.49 |
| FB^n^/F^n^ | 16.34 (17.93, 19.69) | 16.04 (18.96, 23.22) | -1.10 | 0.27 |
| FB^n^/F^n total^ | 14.05 (15.2, 16.45) | 13.83 (15.94, 18.84) | -1.10 | 0.27 |
| F^n^/(B^n^ + FB^n^) | 4.95 (5.46, 5.99) | 4.22 (5.17, 6.06) | -1.07 | 0.28 |
| B^n^/(F^n^ + FB^n^) | 2.93 (3.66, 4.8) | 3.13 (3.77, 4.65) | -0.21 | 0.83 |
| FBG2^n^/FG2^n^ | 0.13 (0.15, 0.19) | 0.13 (0.14, 0.18) | -0.59 | 0.55 |
| FBG2^n^ /(FG2^n^ + FBG2^n^ ) | 11.62 (13.17, 15.63) | 11.23 (12.6, 15.29) | -0.59 | 0.55 |
| FG2^n^/(BG2^n^ + FBG2^n^) | 4.53 (5.49, 6.26) | 4.85 (5.61, 6.57) | -0.79 | 0.43 |
| BG2^n^/(FG2^n^ + FBG2^n^) | 20.62 (27.39, 35.49) | 20.01 (25.12, 32.05) | -1.20 | 0.23 |
| Fucosylation | 94.16 (95.08, 96.06) | 94.32 (95.24, 95.97) | -0.48 | 0.63 |
| Bisecting GlcNAc | 15.93 (17.03, 18.29) | 15.72 (17.82, 20.26) | -1.35 | 0.18 |
| Sialylation | 17.69 (19.49, 22.26) | 17.97 (20.1, 22.79) | -0.37 | 0.71 |
| Galactosylation |  |  |  |  |
| G0 | 30.08 (36.11, 41.63) | 28.71 (34.69, 38.92) | -0.95 | 0.34 |
| G1 | 30.39 (32.11, 33.57) | 30.92 (32.68, 33.77) | -1.08 | 0.28 |
| G2 | 9.53 (11.44, 14.47) | 10.37 (12.8, 14.63) | -1.01 | 0.31 |

GP, glycan peak; G0, agalactosylation; G1, monogalactosylation; G2, digalactosylation; Mann-Whitney U Test was used.


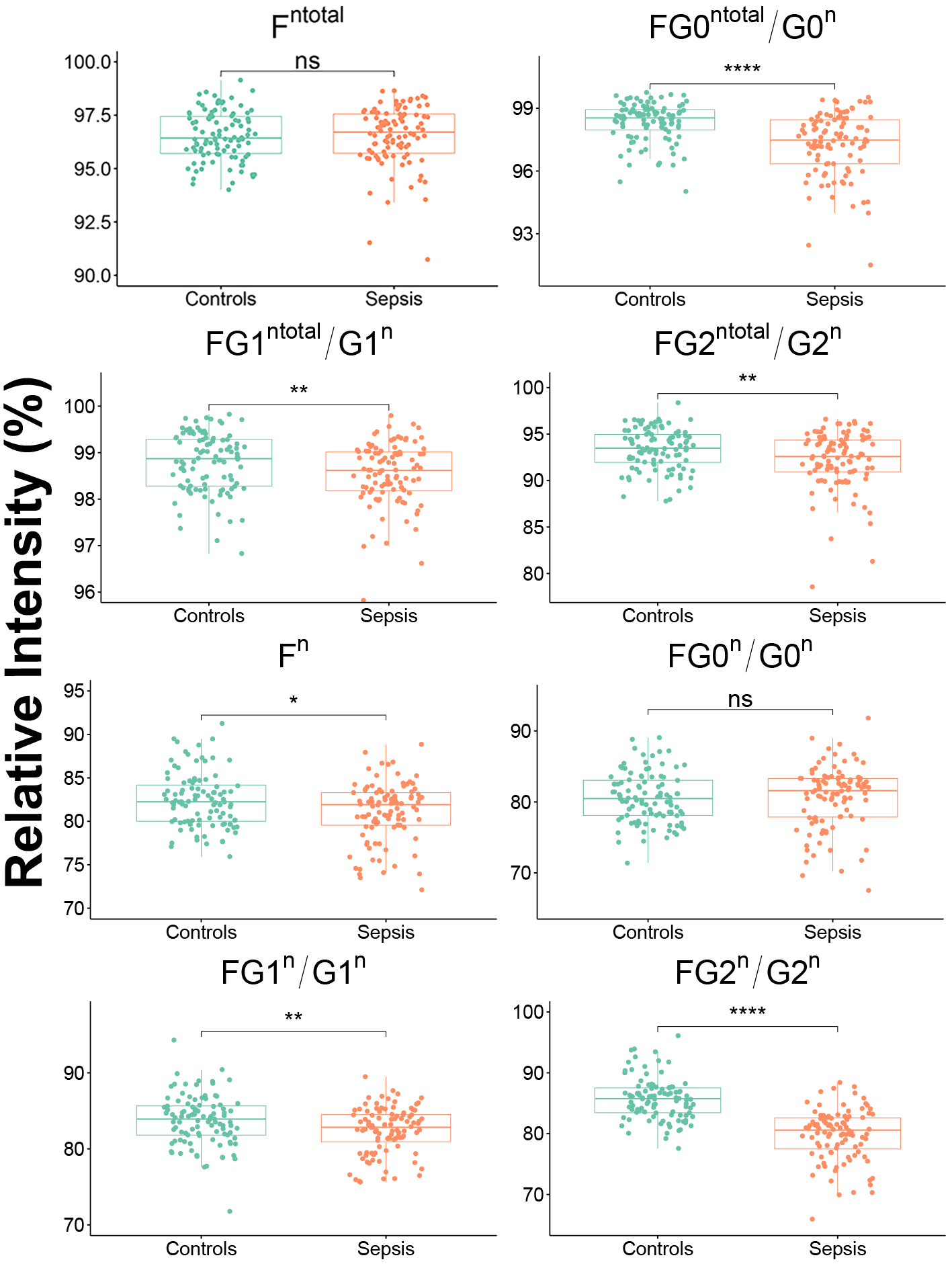


Figure S1 The level of fucosylation in controls and septic patients.

Mann-Whitney U Test was used.


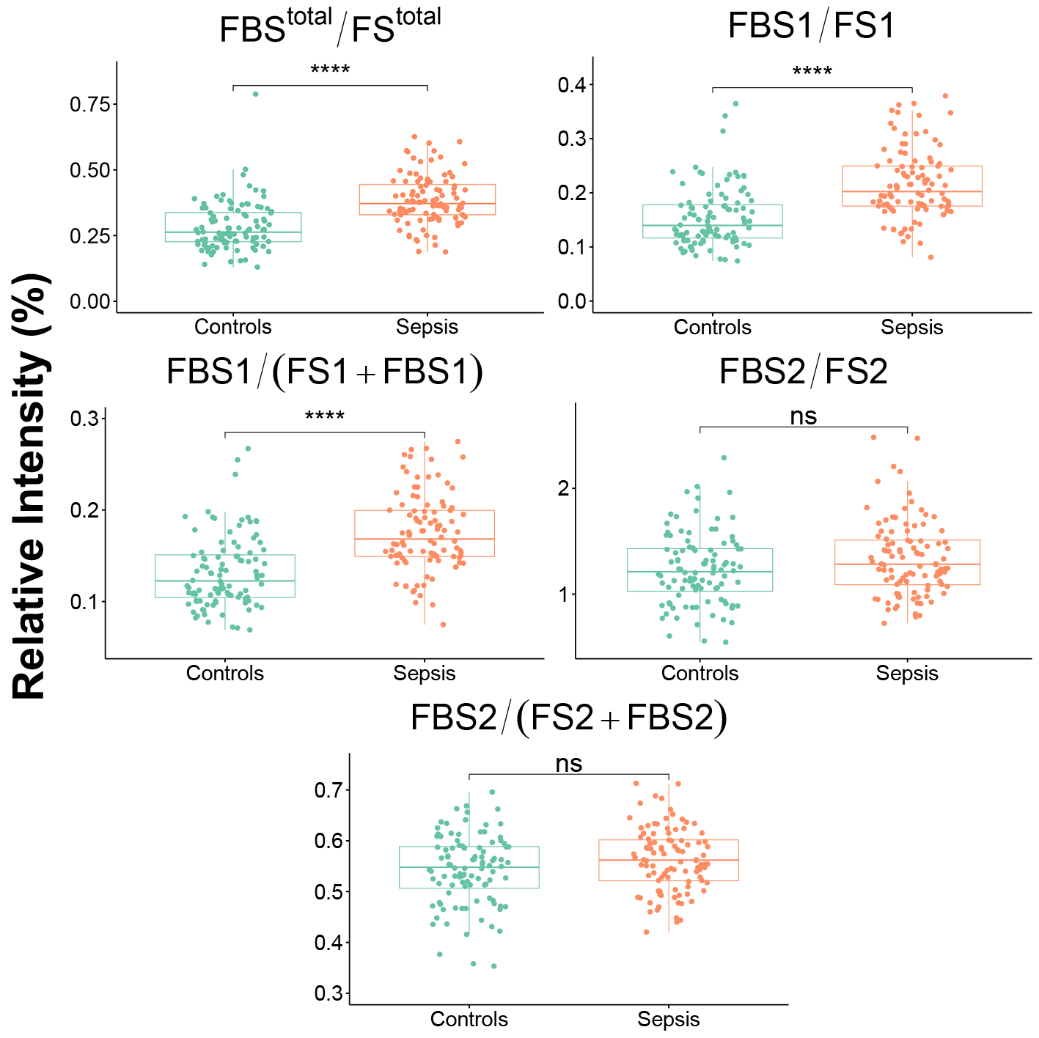


Figure S2 The level of bisecting GlcNAc in controls and septic patients.

Mann-Whitney U Test was used.


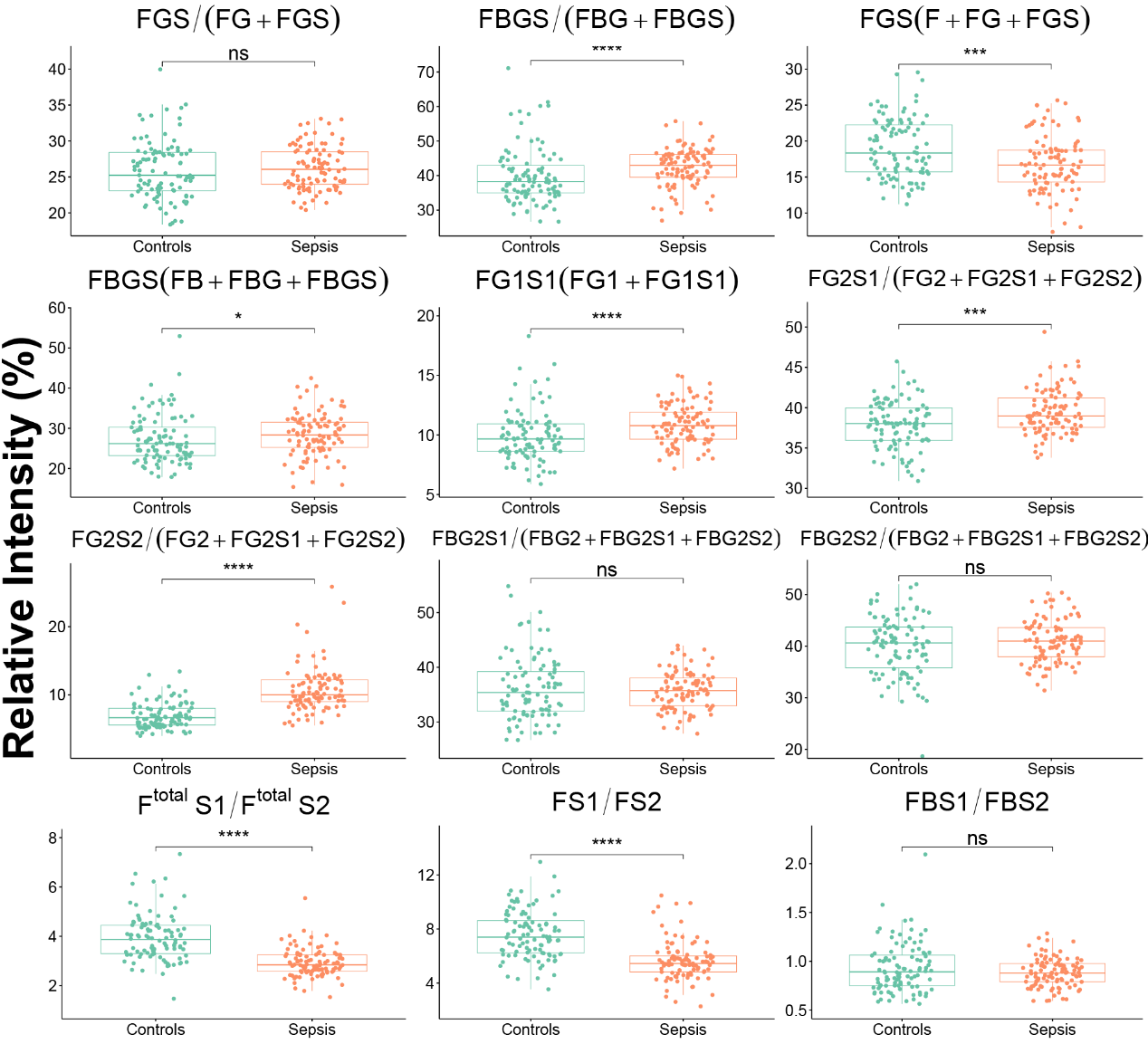


Figure S3 The level of sialylation in controls and septic patients.

Mann-Whitney U Test was used.


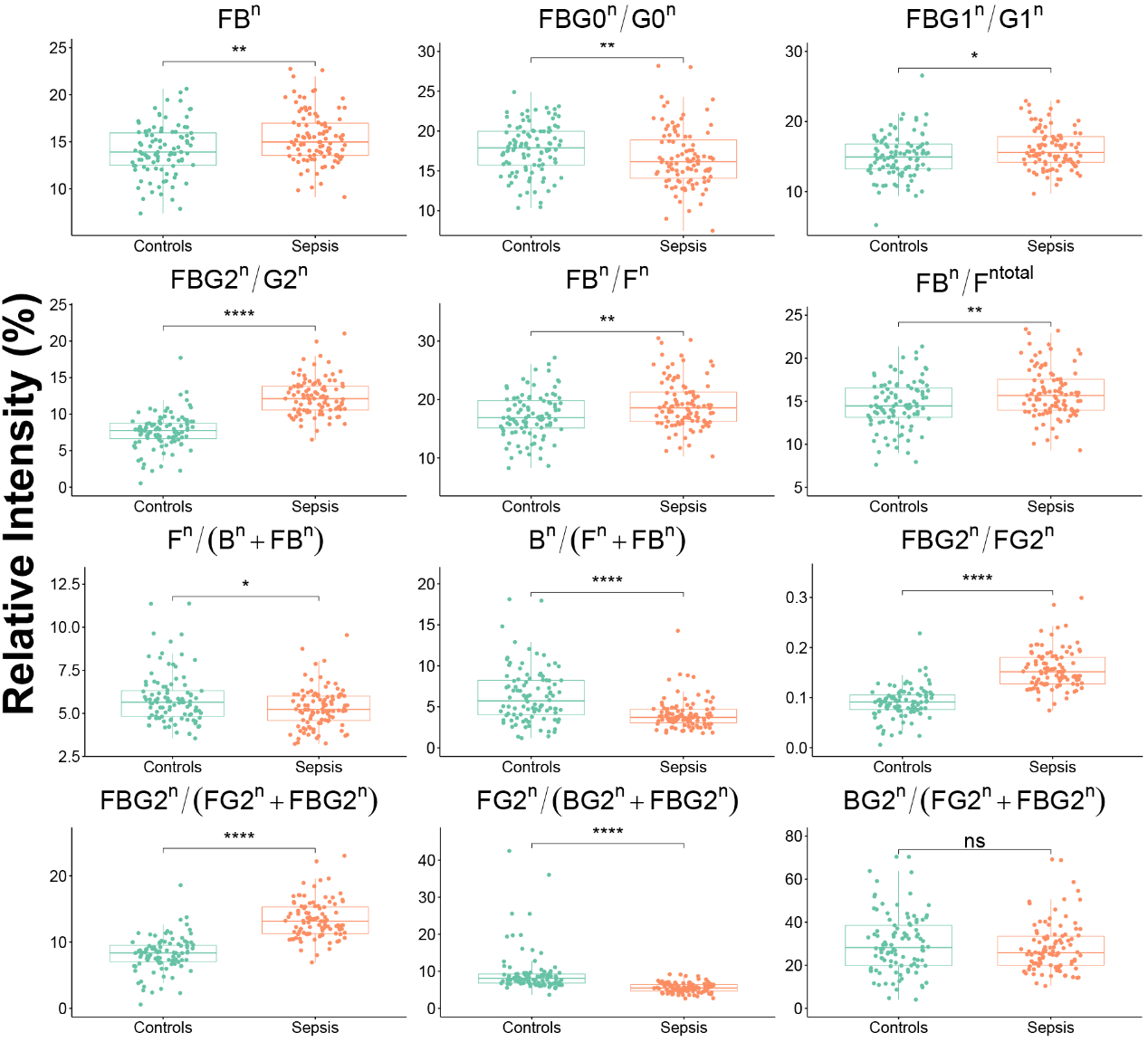


Figure S4 The level of fucosylation with sialylation in controls and septic patients.

Mann-Whitney U Test was used.


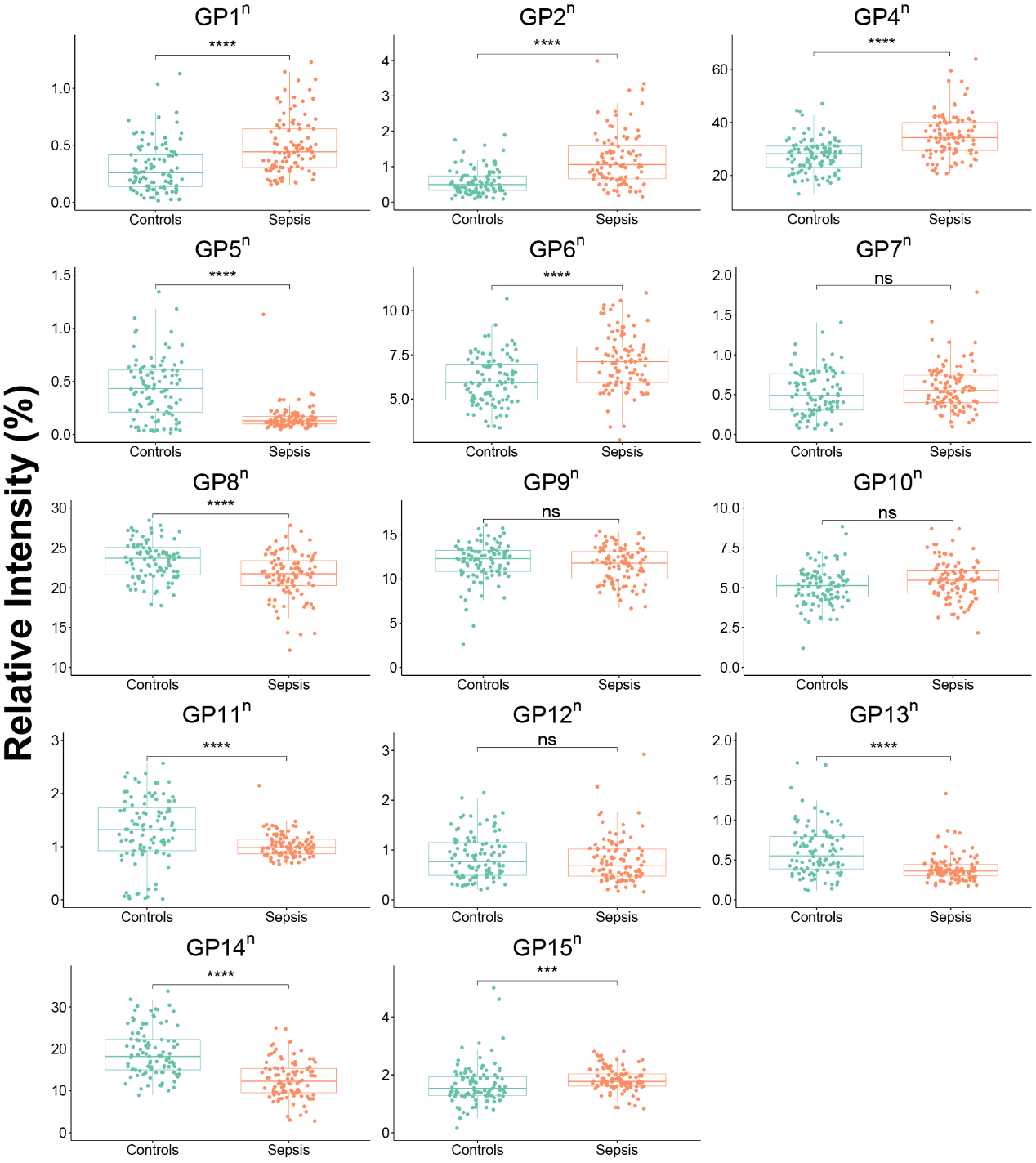


Figure S5 The level of neutral glycans in controls and septic patients.

Mann-Whitney U Test was used.


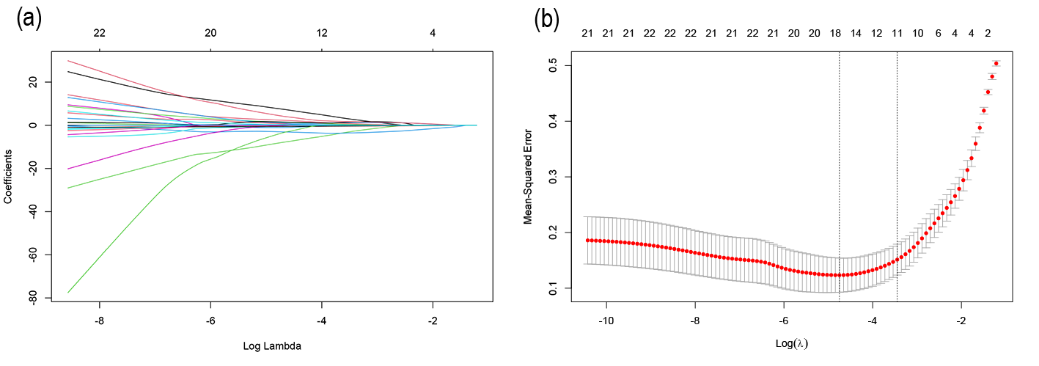


Figure S6 Schematic diagram of the LASSO regression variable shrinkage screening process in population with sepsis.


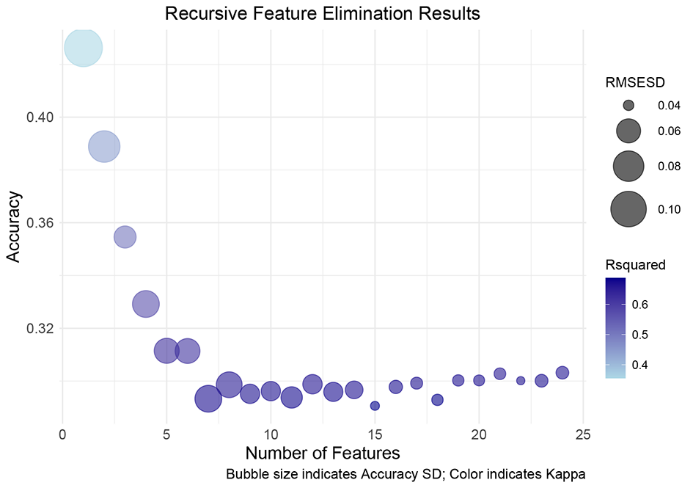


Figure S7 The bubble chart of RFE in population with sepsis.
